# Increased expression of blood muscarinic receptors in patients with reflex syncope

**DOI:** 10.1371/journal.pone.0219598

**Published:** 2019-07-18

**Authors:** Maxime Beutelstetter, Angelo Livolsi, Hugues Greney, Pauline Helms, Catherine Schmidt-Mutter, Charlie De Melo, Gerald Roul, Florian Zores, Alexandre Bolle, Nassim Dali-Youcef, Magali Beaugey, Alban Simon, Nathalie Niederhoffer, Jacques Regnard, Malika Bouhaddi, Chris Adamopoulos, Mickael Schaeffer, Erik Sauleau, Pascal Bousquet

**Affiliations:** 1 Clinical Investigation Center, INSERM 1434, University Hospital of Strasbourg, Strasbourg, France; 2 Biopathology of Myelin, Neuroprotection and Therapeutic Strategies, INSERM U1119, University of Strasbourg, Faculty of Medicine, Strasbourg, France; 3 Unit of Cardiopediatrics, University Hospital of Strasbourg, Strasbourg, France; 4 Laboratory of Neurobiology and Cardiovascular Pharmacology, Federation of Translational Medicine, University of Strasbourg, Faculty of Medicine, Strasbourg, France; 5 Unit of Neonatal Intensive Care, University Hospital of Strasbourg, Strasbourg, France; 6 Unit of Cardiology, University Hospital of Strasbourg, Strasbourg, France; 7 Specialized Medical Group–The Premium, Strasbourg, France; 8 Laboratory of Biochemistry and Molecular Biology, University Hospital of Strasbourg, Strasbourg, France; 9 Institute of Genetics and Molecular and Cellular Biology, Department of Functional Genomics and Cancer, Illkirch, France; 10 Physiology-Functional Explorations, Regional University Hospital of Besançon, Besançon, France; 11 Department of Public Health, methods in clinical research, University of Strasbourg, Strasbourg, France; Weizmann Institute of Science, ISRAEL

## Abstract

**Aims:**

Pathophysiology of reflex syncope is not fully understood but a vagal overactivity might be involved in this syncope. Previously, overexpression of muscarinic M_2_ receptors and acetylcholinesterase was found in particular in the heart and in lymphocytes of rabbits with vagal overactivity as well as in hearts of Sudden Infant Death Syndromes. The aim of this present study was to look at M_2_ receptor expression in blood of patients with reflex syncope. The second objective was to measure acetylcholinesterase expression in these patients.

**Methods and results:**

136 subjects were enrolled. This monocenter study pooled 45 adults exhibiting recurrent reflex syncope compared with 32 healthy adult volunteers (18–50 years) and 38 children exhibiting reflex syncope requiring hospitalization compared with 21 controls (1–17 years). One blood sample was taken from each subject and blood mRNA expression of M2 receptors was assessed by qRT-PCR. Taking into account the non-symmetric distributions of values in both groups, statistical interferences were assessed using bayesian techniques. A M_2_ receptor overexpression was observed in adult and pediatric patients compared to controls. The medians [q1;q3] were 0.9 [0.3;1.9] in patients *versus* 0.2 [0.1;1.0] in controls; the probability that M_2_ receptor expression was higher in patients than in controls (Pr[patients>controls]) was estimated at 0.99. Acetylcholinesterase expression was also increased 0.7 [0.4;1.6] in patients *versus* 0.4 [0.2;1.1] in controls; the probability that acetylcholinesterase expression was higher in patients than in controls (Pr[patients>controls]) was estimated at 0.97. Both in adults and children, the expression ratio of M_2_ receptors over acetylcholinesterase was greater in the patient group compared with the control group.

**Conclusion:**

M_2_ receptor overexpression has been detected in the blood of both, adults and children, exhibiting reflex syncope. As in our experimental model, *i*.*e*. rabbits with vagal overactivity, acetylcholinesterase overexpression was associated with M_2_ receptor overexpression. For the first time, biological abnormalities are identified in vagal syncope in which only clinical signs are, so far, taken into account for differential diagnosis and therapeutic management. Further work will be needed to validate potential biomarkers of risk or severity associated with the cholinergic system.

## Introduction

Syncope is defined by the ACC/AHA/HRS Syncope Guideline as “an abrupt, transient, complete loss of consciousness and postural tone, secondary to inadequate cerebral perfusion” [1]. The onset of syncope is relatively rapid, and the subsequent recovery is spontaneous, complete, and relatively prompt. It is a common pathology, representing around 5% of hospital admissions, 3% of emergency unit visits [2] and 1 to 3% of pediatrics emergency department visits [3]. Despite their brief and benign nature, frequent reflex syncope can influence the quality of life of patients [4] and reflex syncope in children is a source of stress for their parents. They may be anxious and consider that their child has either stopped breathing and many will pick their child up or attempt cardiopulmonary resuscitation, or both [5].

Reflex (or neutrally-mediated) syncope is defined as “a syncope due to a reflex that causes vasodilation, bradycardia or both” [1]. Vasovagal syncope, the most common form of reflex syncope mediated by the vasovagal reflex and associated to cerebral hypoperfusion and triggered by emotion or orthostatic stress, is usually preceded by prodromal symptoms of autonomic system activation (sweating, pallor, or nausea) [1,6]. Vagal overactivity (VO), with an inappropriate reflex implicating afferent, central, and efferent pathways, seems to be involved in the reflex syncope, particularly in vasovagal syncope [7]. The vagal nervous system is considered as having a protective role [8], balancing the sympathetic influence on the cardiac rhythm [7]. The cardiac vagus influences heart function through the sinus node and also through intraventricular parasympathetic fibers which are likely to add their effects on those of the fibers terminating in the sinus node [9]. In addition, there are muscarinic receptors in cardiomyocytes [10].

So far, no biological abnormalities were identified in reflex syncope. Our hypothesis is that biological abnormalities of the cholinergic system are associated with clinical manifestations of VO such as reflex syncope. The biological substrate of a VO could be i) exaggerated production and release of the neurotransmitter, acetylcholine; ii) abnormally high density of muscarinic M receptors (M_2_R) specific to acetylcholine; iii) abnormally low enzymatic inactivation of acetylcholine by the acetylcholinesterase (AchE). The measurement of acetylcholine in real time is neither easy nor reliable. Thus, we tested the hypothesis of muscarinic receptor overexpression, first in an experimental model of vagal hyperactivity [11] and an abnormally high muscarinic receptor density was observed in the myocardium of rabbits presenting VO [11,12]. We have observed a good correlation between the muscarinic receptor density and the ECG R-R intervals, *i*.*e*. inverse correlation with the heart rate. This observation concerned the M_2_ and M_3_ subtypes of muscarinic receptors expressed in the heart of rabbits. But it was for the M_2_ receptors (M_2_R) that the correlation was the best [12]. For the rest of our exploratory work in this area, we focused on the M_2_ subtype. For our work in humans, we had one more reason to focus on this subtype; it is the subtype mainly expressed in the human myocardium [13]. M_2_R overexpression was most often accompanied by an unexpected increase of AchE expression or activity [12,14]. This AchE abnormality was interpreted as a possible compensatory response to M_2_R overexpression. Both abnormalities were found in the white blood cells of the rabbit VO model [12]. Muscarinic receptors, including M_2_R, are expressed in lymphocytes and their mRNAs were detected in the majority of mononuclear immune cells with a significant variability among subjects [15,16]. So far, no plasma M_2_R have been identified. M_2_R overexpression and AchE overexpression were also observed in hearts of infants deceased from Sudden Infant Death Syndrome (SIDS) [14]_._

The present study aimed at testing whether M_2_R overexpression could be associated with VO in a relatively common clinical situation, namely reflex or neutrally-mediated syncope, usually uncomfortable in adults and possibly harmful in children [17].

## Methods

### Study design

This study was a monocenter, pathophysiological, and comparative study. Adult patients exhibiting recurrent reflex syncope, *i*.*e*. at least 3 episodes, were compared to healthy adult volunteers. Children exhibiting reflex syncope requiring hospitalization or medical consultation were compared to hospitalized children with neither history of syncope nor cardiac pathology. The adult and children subjects were recruited from the Clinical Investigation Center, adult cardiology unit, and pediatrics unit.

### Study approval

This study was performed in accordance with the principles of the Declaration of Helsinki and the guidelines for Good Clinical and Laboratory Practice. The study was approved by the institutional review board of the Comité de Protection des Personnes-Est IV, Strasbourg, France (N° IDRCB 2010-A01451-38) and recorded in ClinicalTrials.gov (NCT01358461). The authors designed and conducted the study, managed the database, and wrote all drafts of the manuscript. All the authors vouch for the accuracy and completeness of the data and reported analyses, as well as for the study’s adherence to the protocol.

### Subjects

We recruited 136 subjects in this study at the University Hospital of Strasbourg. All subjects met the inclusion and exclusion criteria (Table 1) and completed all assessments. 83 patients with reflex syncope were compared to 53 control subjects with no current or history of syncope. Pediatric patients, 38 children; age range: [1 to 18 years], were enrolled in pediatric unit after medical consultation for reflex syncope. 21 control children were selected in the cardio-pediatric unit after examination of medical history. 45 Adult patients [18 to 50 years] were enrolled in the cardiology unit after medical consultation for recurrent reflex syncope. 32 healthy adult volunteers were recruited for control adult group. All adult subjects and the parents or guardians of the minors were informed orally and writing of the study’s modalities and signed consent. Adult subjects received a financial compensation for this study.

**Table 1:** Detailed inclusion and exclusion criteria for enrollment in the study.

	Inclusion criteria	Exclusion criteria
**Adult patients**	Men or women aged 18 to 50 Patients with clinical signs of recurrent reflex syncope (>3 episodes) Normal electrocardiogram Normal blood count No contraindication for the carotid sinus massage test in carotid Doppler echography Non-smoking or occasional smoking (<3 per day)	Cardiovascular, neurological, psychiatric, immunologic, infectious or metabolic diseases Dysautonomia Ongoing treatment targeting the autonomic nervous system Consumption of doping agents or addiction (cocaine, cannabis, amphetamines, alcohol…) Consumption of tobacco, coffee, within the 24 hours before the clinical and laboratory investigations Pregnancy or breastfeeding
**Adult controls**	Men or women aged 18 to 50 No history of reflex syncope or cardiac diseases Normal electrocardiogram Normal blood count No contraindication for the carotid sinus massage test in carotid Doppler echography Non-smoking or occasional smoking (<3 per day)	
**Pediatric patients**	Males or females aged 1 to 18 Patients with clinical signs of reflex syncope requiring hospitalization or medical consultation Normal electrocardiogram Normal blood count Non-smoking or occasional smoking (<3 per day)	
**Pediatric controls**	Males or females aged 1 to 18 No history of reflex syncope or cardiac diseases Normal electrocardiogram Normal blood count Non-smoking or occasional smoking (<3 per day)	

### Study endpoints

The primary endpoint was M_2_R mRNA expression in the blood of adults and children exhibiting reflex syncope. The secondary endpoint was AchE mRNA blood expression.

### Study assessments

The selection visit was conducted by a cardiologist, involving clinical examinations, medical history, current treatment, electrocardiogram, and echo-Doppler of carotid arteries (for subjects over 40 years of age). During the following visit, Holter electrocardiographic monitoring was carried out in all adult subjects (see S1 File for assessment details and interpretation criteria). Holter electrocardiographic, usually performed in case of recurrent syncope, was considered positive (significant vagal overactivity) when squared differences between adjacent normal RR intervals (RMSSD) were higher than 56 milliseconds (ms) in males and 62ms in females and when the percentage of adjacent intervals over 50 ms (PNN50) was higher than 28% in males and 29% in females [18,19]. A carotid sinus massage test (CSMT) was also performed in adult subjects. In this study the CSMT was performed by manually massaging for 5 to 10 seconds the anterior sternocleidomastoid muscle on a motorized inclination table (Genin-France) with continuous blood pressure blood monitoring (in accordance with the FinapressTM instructions) and the heart rate (Task Force Monitor, CNS system, Graz, Austria). CSMT was suggestive of exaggerated parasympathetic activity when the heart rate reduction in response to carotid massage was greater than 10% or the mean arterial pressure decreased by more than 20% compared to the baseline [20].

Two blood samples were collected from each subject for the complete blood count and mRNA specific analysis (M_2_R and AchE). For the complete blood count, 4mL of peripheral blood were sampled. Assessments were performed in the hematology laboratory of Strasbourg University Hospital. The second blood sample (2.5mL) was collected in PaxGene Blood RNA Tubes according to Qiagen recommendations. Total RNA was extracted automatically by Qiacube with the PaxGene RNA kit (Qiagen); 200ng of total RNA were reverse transcribed into cDNA, using the iScript cDNA Synthesis kit (Biorad). Target gene expressions were measured by quantitative real-time polymerase chain reaction (qRT-PCR) using a specific primer for the target genes: CHRM2 for M_2_R and ACHE for AchE. The relative concentrations of the target genes were calculated using a standard range created from a pool of 70 RNA of healthy subjects. The housekeeping gene (HKG) ribosomal 18S RNA was employed for the normalization of the expressions [21,22], the same as that used in our previous studies for the same purpose [12,14]. M_2_R and AchE expressions normalized by 18S are expressed as arbitrary units (a.u.). The samples were analyzed in a blinded procedure. Detailed laboratory data are provided as Supporting Information, S1 Methods.

### Statistics

The initial characteristics of patients were described in each group by means and standard error of the mean or effectives, depending on the nature of each variable: quantitative or categorical. Results were expressed as median with 25 and 75 quantiles (also in box plot graphs) for continuous variables or number of individuals (and frequency) of each modality for qualitative variables.

To model the studied normalized values, *e*.*g*. M_2_R:18S, in both groups, generalized linear models with Gamma distribution (and a logarithmic link function) were used, for dealing with strictly positive yet asymmetric values, thus attributing less weight to extreme values. More specifically, the mean of a normalized value was explained additively by a grand mean and a group effect. Statistical inference was carried out using Bayesian technics [23].

Markov Chain Monte Carlo (MCMC) methods with one chain, 5,000 burn-in iterations and 100,000 iterations were used for generating samples of posterior distributions. Estimate for the model coefficients have been presented as mean and [95% credible interval] from each posterior distribution. Convergence of the chains was checked in every case. Minimally informative prior distributions were chosen as normal distribution with zero mean and a variance of ten for the parameters in regression models [23]. The probability for the group parameter to be strictly positive, *i*.*e*. the probability of observing a positive difference between groups, was estimated from the quantiles of its posterior distribution. According to the context of this study, such a probability greater than 0.90 was considered to be strongly relevant. All analyses were performed using R software [24].

## Results

The characteristics of the subjects have been presented in Table 2. All detailed results are available in Supporting Information.

**Table 2:** Characteristics of the study subjects.

		Patients		Controls	
		Adults	Children	Adults	Children
**Number of subjects**		45	38	32	21
**Total**		83		53	
**Number of subjects by gender**	**Females**	38	20	12	8
	**Total**	58		20	
	**Males**	7	18	20	13
	**Total**	25		33	
**Age in inclusion (mean +/- SEM years)**		27·3 ± 8·6	8·8 ± 4·8	24·8 ± 5·4	7·7 ± 3·6
**Total (mean +/- SEM years)**		18·9 ± 11·7		18·0 ± 9·7	
**Carotid Sinus Massage Test**	Positive*	34	NA	8	NA
	**Negative**	10		24	
**Holter electrocardiographic monitoring**	**Positive†**	16	NA	6	NA
	**Negative**	28		26	

SEM: standard error of the mean

*: CSMT was suggestive of exaggerated parasympathetic activity, when the heart rate reduction in response to carotid massage was greater than 10% or mean arterial pressure decreased by more than 20% compared to the baseline.

†: A Holter test was considered positive (significant vagal activity) when rMSSD was higher than 56ms in males and 62ms in females and when PNN50 was higher than 28% in males and 29% in females.

### M_2_R and AchE expression in the pooled groups of subjects (adults and children) (Fig 1)

**Fig 1:**
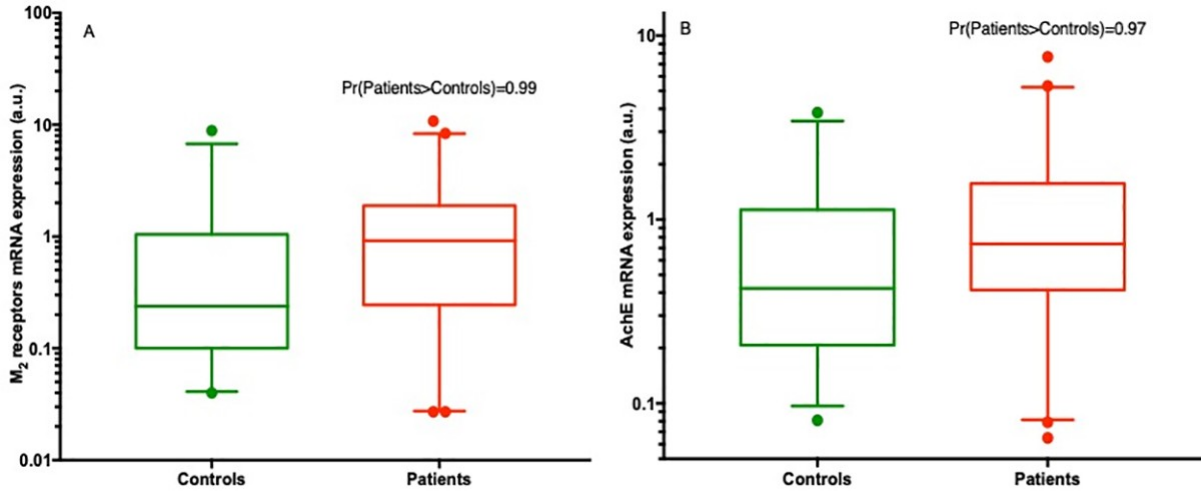
mRNA expression of muscarinic M_2_ receptors (M_2_R), acetylcholinesterase (AchE) and ratio of M_2_R/AchE mRNA expression in the pooled groups of subjects (on log-scale). **A**:Medians of mRNA M_2_R expression with 25 and 75 percentiles in box plots based on all subject data, along with the probability that M_2_R expression is greater in the patient group than the control group [Pr(patients>controls)], estimated from the posterior distribution in regression models **B:** Medians of mRNA AchE expression with 25 and 75 percentiles in box plots based on all subject data, and the probability that AchE expression is greater in the patient group than the control group [Pr(patients>controls)], estimated from the posterior distribution in regression models.

The medians [q1; q3 interval] of M_2_R expression level were 0.2 [0.1; 1.0] in the control group and 0.9 [0.3; 1.9] in the patient group, including children and adults. The probability that M_2_R expression was greater in the patient group than in the control group was estimated at Pr[patients >controls] = 0.99 in this group (Fig 1A).

The medians of AchE expression were 0.4 [0.2; 1.1] in the control group and 0.7 [0.4; 1.6] in the patient group, with probability of superiority Pr[patients >controls] = 0.97 (Fig 1B).

### M_2_R:AchE expression ratio (S1 Fig)

The medians of the M_2_R:AchE ratio were 0.8 [0.5; 1.1] in the control group and 1.1 [0.8; 1.3] in the patient group. The probability that the M_2_R:AchE expression ratio was greater in the patient group than in the control group was estimated at Pr[patients >controls] = 1.00 for all the patients.

### Correlations

No strong correlations were found between the M_2_R expression and the number of syncope (measured by the lifetime cumulative events of syncope), age of subject or gender. The same applied for AchE expression.

### M_2_R and AchE mRNA expression in the adult group (Fig 2)

**Fig 2:**
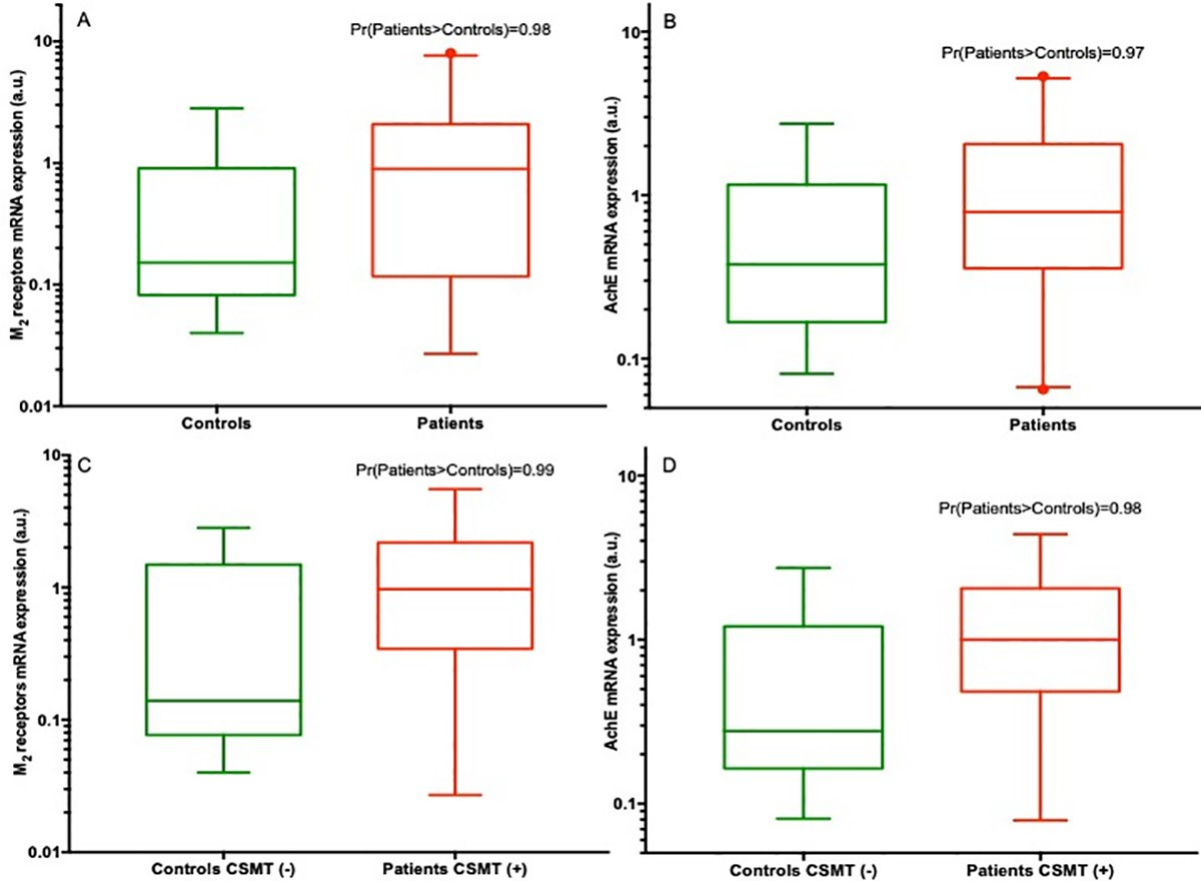
Expressions of M_2_R and AchE in the adult group (on log-scale). **A**: Medians of mRNA M_2_R expression with 25 and 75 percentiles in box plots based on adult group data, along with the probability that M_2_R expression is greater in the patient group than the control group [Pr(patients>controls)], estimated from the posterior distribution in regression models **B**: Medians of mRNA AchE expression with 25 and 75 percentiles in box plots based on adult group data, and the probability that AchE expression is greater in the patient group than the control group [Pr(patients>controls)], estimated from the posterior distribution in regression models **C**: Medians of mRNA M_2_R expression with 25 and 75 percentiles in box plots based on adult group data, along with the probability that M_2_R expression is greater in the patient group than the control group [Pr(patients>controls)], estimated from the posterior distribution in regression models. The results of the Carotid Sinus Massage Test (CSMT) are considered: CSMT(-) for a negative test and CSMT (+) for a positive test. **D**: Medians of mRNA AchE expression with 25 and 75 percentiles in box plots based on adult group data, and the probability that AchE expression is greater in the patient group than the control group [Pr(patients>controls)], estimated from the posterior distribution in regression models. The results of the Carotid Sinus Massage Test (CSMT) are considered: CSMT(-) for a negative test and CSMT (+) for a positive test.

When adults were analyzed separately, we found very similar results. In brief, the probability that M_2_R expression was greater in the patient group 0.9 [0.1; 2.0] than in the control group 0.2 [0.1; 0.8] was estimated at Pr[patients >controls] = 0.98 (Fig 2A).

The medians of AchE expression were 0.8 [0.4; 2.1] in the patient group and 0.4 [0.2; 1.1] in the control group, with a probability of superiority for the patients estimated at Pr[patients >controls] = 0.97 in the adult patients (Fig 2B).

### M_2_R:AchE expression ratio in the adult group (S2 Fig)

The probability that the M_2_R: AchE expression ratio was greater in the patient group than in the control group was estimated at Pr[patients >controls] = 0.98 in adults. The medians were 1.1 [0.7; 1.3] and 0.8 [0.5; 0.9] in patients and controls, respectively.

### Carotid sinus massage test and Holter electrocardiographic monitoring (Fig 2)

When including the carotid sinus massage test (CSMT) data, the results were even more pronounced. The probability that M_2_R mRNA expression was greater in the patient group with positive CSMT (1.0 [0.4; 2.1]) than in the control group with negative CSMT (0.1 [0.1; 1.1]) was estimated at Pr[patients >controls] = 0.99. As for AchE expression, this probability was estimated at 0.98. The medians were 1.0 [0.5; 1.9] in the patient group (with positive CSMT) and 0.3 [0.2; 1.2] in the control group (with negative CSMT) (Fig 2C and 2D).

As for the Holter electrocardiographic monitoring results, alone or with CSMT results, the probabilities were lower (detailed results in Supporting Information).

### M_2_R and AchE mRNA expression in the pediatric group (Fig 3)

**Fig 3:**
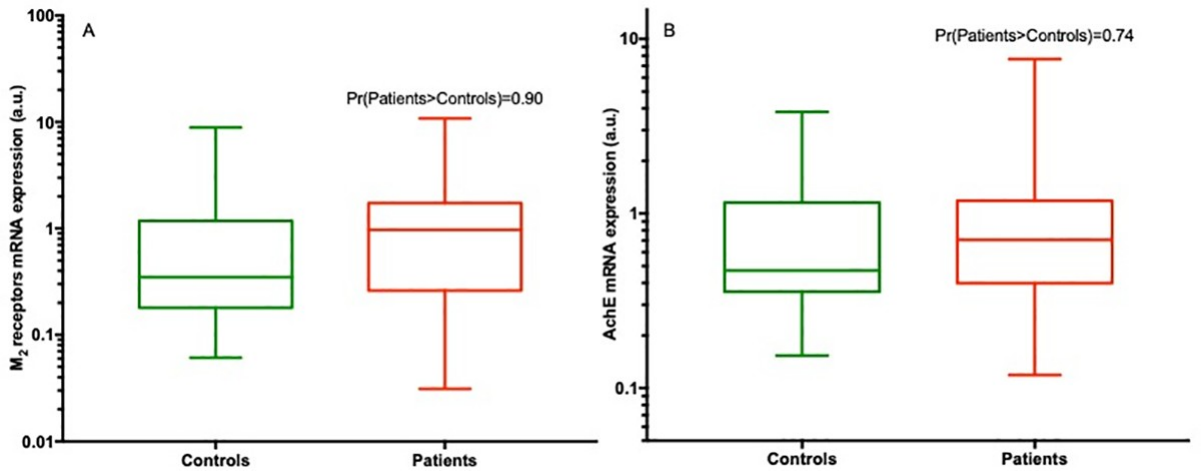
Expressions of M_2_R and AchE in the pediatric group (on log-scale). **A**: Medians of mRNA M_2_R expression with 25 and 75 percentiles in box plots based on pediatric group data, along with the probability that M_2_R expression is greater in the patient group than the control group [Pr(patients>controls)], estimated from the posterior distribution in regression models **B**: Medians of mRNA AchE expression with 25 and 75 percentiles in box plots based on pediatric group data, and the probability that AchE expression is greater in the patient group than the control group [Pr(patients>controls)], estimated from the posterior distribution in regression models.

In children, the probability that M_2_R expression was greater in the patient group 1.0 [0.3; 1.6] than in the control group 0.3 [0.2; 1.1] was estimated at Pr[patients >controls] = 0.90 (Fig 3A).

The medians of AchE expression were 0.7 [0.4; 1.1] in the patient group and 0.5 [0.4; 1.1] in the control group, with a probability for superiority in the patient group in the pediatric group Pr[patients >controls] = 0.74 (Fig 3B).

### M2R: AchE expression ratio in the pediatric group (S3 Fig)

The probability that the M_2_R:AchE expression ratio was greater in the patient group 1.2[0.8; 1.6] than in the control group (1.1[0.6; 1.3]) was estimated at Pr[patients >controls] = 0.97.

## Discussion

From the beginning, we considered the prospect of future development of a blood marker, and for this purpose, we relied on the studies of the Kawashima's group, demonstrating the existence of a complete cholinergic system, including muscarinic receptors and AchE, in lymphocytes. In addition, our first studies in rabbits and in cases of SIDS showed the similarity between what we observed in the heart and in nuclear blood cells [12,14].

According to our previous studies, all types of muscarinic receptors seem to be increased in blood; in this study, we focused our research in the M_2_ subtype, which is mostly expressed in the heart [12]. M_2_R mRNA was largely overexpressed in the child and adult patients compared to the controls. These findings confirm the study’s primary objective. AchE mRNA was also increased in patients compared to controls, although greater difference was observed in adults than in children. There was no correlation between M_2_R or AchE expression and age, gender or number of syncope.

It is important to note that two previous studies from our team, have shown that mRNA expression (qRT-PCR) and protein expression (binding experiments) follow parallel changes [12,14]. In other words, mRNA expression clearly results in protein translation in the case of M_2_R proteins. Therefore, using qRT-PCR technique to assess M_2_R expression was justified [25]. The same applied for mRNA and AchE expression. The qRT-PCR is an accurate and sensitive method to quantify gene expression. To reduce factors which can diminish RT-PCR accuracy, *e*.*g*. quality of RNA, cDNA synthesis by reverse transcriptase, PCR amplification efficiencies, reference genes, known as HKG, are used as internal controls for normalizing the relative expression of target genes [21,22]. In this study, we used the 18S ribosomal gene as a HKG, which was used previously [12,14,17]. Our previous studies have also shown a correlation between cardiac M_2_R density and M_2_R mRNA expression in blood, the same overexpressions were found in heart and in blood [12]. Based on this background and for practical reasons, the mRNA expression was assessed in the blood of all the subjects. We took 2.5mL whole blood samples using PaxGene tubes that enabled rapid and easy analysis.

It is interesting to note that in our study M_2_R overexpression was assessed in blood, confirming that the muscarinic dysfunction is not restricted to the heart as previously reported [12,14]. As mentioned above, all elements of a cholinergic system are expressed in lymphocytes, *i*.*e*. synthesis and degradation enzymes of acetylcholine and various muscarinic receptors [15,16]. But it is possible that, in addition, there might be interactions between peripheral muscarinic receptors and the vagal system that remain to be explored. Our results suggest that, subject to validation, M_2_R overexpression could become a biological marker of VO.

In this exploratory work, we aimed to exhaustively measure the expression of different forms of AchE and to do so we designed oligonucleotides common for all three isoforms of AchE (E,S and R) [26,27]. We experimentally measured mRNA expression in white blood cells (WBC) and eventually that of red blood cells (RBC). We employed the HKG ribosomal 18S for normalization; therefore, the mRNA expression in WBC (or eventually RBC) does not vary with their number. Future studies could be devoted to the expression of the 3 AchE variants. The significant AchE overexpression observed, at least in adults, strengthens the hypothesis that this is a compensatory mechanism secondary to M_2_R overexpression itself. In children, AchE was also overexpressed, albeit to a lesser extent than in adults, suggesting that the above-mentioned compensatory mechanism is not fully mature at that development stage, whereas M_2_R expression abnormality occurs very early in life [14,17].

In our patients, the average M_2_R/AchE expression ratio was higher than in controls for the total subjects, as well as in adult and pediatric subgroups. These ratio differences indicate that the increase in M_2_R expression is more relevant than that of AchE. These results suggest that, in patients, the compensatory increase of AchE expression could be insufficient to prevent syncope. Finally, the combination of M_2_R overexpression and incomplete compensation by AchE overexpression might be the mechanism behind reflex syncope, particularly the very severe equivalent of reflex syncope in infants [17].

In some situations, particularly in infants, the expression of M_2_R can be highly increased whereas the increase of the enzyme expression is not as important [17]; therefore, the hypothesis according to which the overexpression of the AchE could be the primary phenomenon which in turn would induce an upregulation of the M_2_R can be precluded. We propose this compensatory mechanism as a hypothesis. Biologically, it remains to demonstrate that the increase in the expression of M_2_R and that of AchE originate in the same cell type. In any case, our results show that, at least on the functional point of view, there is a compensatory association, complete or partial according to the clinical situations, between the two phenomena. As in children especially those with iALTE, the overexpression of the AchE is much less than that of the M_2_R, it is tempting to assume that the overexpression of the AchE is sometimes insufficient to compensate that of M_2_R. Our results are compatible with the hypothesis of a systemic dysfunction of the cholinergic system in this pathology.

As already mentioned in the introduction, our results do not exclude that a sympathetic dysregulation can be the primary cause of the dysfunction of the sympatho-vagal balance [28]. But our results support the hypothesis according to which VO could be the final common pathway of vagal hyperactivity.

Especially in the adult subjects, the blood samples were collected at different times in relation to their last syncope. In some cases, blood has been collected quite a long period afterwards. This could generate variability and thus a loss of power and sensitivity in the dosing of the expression of M_2_R and AchE. Thus, the question of whether these biological abnormalities are a transient rather than a permanent process remains open. To answer this question, a longitudinal study should be conducted to assess these biological parameters at different time intervals after the last syncope.

Carotid sinus massage test (CSMT) is a functional test often used in adults with syncope of unknown origin, but possibly reflex syncope [1]. In our study, a CSMT was systematically performed in all subjects, who did not have any contraindication to assess carotid sinus hypersensitivity associated with vagal parasympathetic overactivity. As a matter of fact, the difference in M_2_R expression between the two adult groups was even more significant when not only clinical criteria but also CSMT data were considered. This observation confirms that CSMT is suitable for testing VO, at least via M_2_R expression assessment, whereas Holter data does not influence the difference. To homogenize our patient selection, we could have used positive CSMT as a selection criterion, in addition to clinical parameters [29]. However, no relationships between CSMT, VO, and M_2_R density had previously been demonstrated. Our findings highlight such relationships and thus indicate that CSMT could be used in future studies in this domain.

### Limitations

Several limitations of our study should be noted. Our results on M_2_R are consistent with a coherent pathophysiology scenario concerning reflex syncope. However, they do not exclude the involvement of other biological parameters, *i*.*e*. neuromediators such as adenosine [30,31], receptors, enzymes, hormones. In other words, the pathophysiology of the neuro-mediated syncope might be more complex than the only overexpression of muscarinic receptors They are mainly located in the cardiac atria and are primarily responsible for slowing the heart rate. Long lasting cardiac pauses as a consequence of the M_2_R overexpression may explain syncope; M_2_R are also expressed in the vascular walls [32]. Whether or not vascular M_2_R overexpression may also contribute to syncope still remains to be investigated.

A quite great inter-individual variability of M_2_R and AchE expression in each group was observed. This variability seems to be due to a certain inaccuracy of the clinical criteria currently used for the diagnosis of vagal syncope and consequently for the definition of the inclusion and exclusion criteria used for patient recruitment in our study. Therefore, future studies on this topic, in particular those aiming at clarifying the association between muscarinic receptor overexpression and vagal pathologies, will likely benefit from greater precision of inclusion and exclusion criteria.

Studies have shown the implication of genetic transmission in reflex syncope [33,34]. So far, whether overexpression of M_2_R or AchE might be due to genetic mutations or secondary to excessive acetylcholine release remains unexplored. A clinical study is yet ongoing to determine the role of genetics in reflex syncope.

## Conclusion

In adults, as in children, M_2_R overexpression appears as a hallmark of VO. Our work helps to understand the pathophysiology of VO. For the first time, biological abnormalities have been identified in pathologies in which, until now, only clinical elements were taken into account for the differential diagnosis and therapeutic management. Further work will be needed to validate potential biomarkers of risk or severity associated with the cholinergic system. This could lead to better management of reflex syncope.

These abnormalities are also observed in other human pathologies, such as SIDS or idiopathic apparent life-threatening event (iALTE) [14,17]. Ours results open the path to forthcoming studies aiming validating M_2_R expression as a biological marker of these pathologies.

## Supporting information

S1 File (DOCX)

S1 Methods Additional methodology details. (DOCX)

S1 Table Detailed data of all subjects. (DOCX)

S2 Table Detailed results of descriptive analyzes for the total population. (DOCX)

S3 Table Detailed results of descriptive analyzes for the adult population. (DOCX)

S4 Table Detailed results of descriptive analyzes for the total population. (DOCX)

S5 Table Detailed results of descriptive analyzes for the pediatric population. (DOCX)

S6 Table Detailed results of inferential analyzes of M2 receptors expression for the total population. (DOCX)

S7 Table Detailed results of inferential analyzes of Acetylcholinesterase expression for the total population. (DOCX)

S8 Table Detailed results of inferential analyzes of M2 receptors: Acetylcholinesterase expressions ratio for the total population. (DOCX)

S9 Table Detailed results of inferential analyzes of M2 receptors expression for the adult population. (DOCX)

S10 Table Detailed results of inferential analyzes of AchE expression for the adult population. (DOCX)

S11 Table Detailed results of inferential analyzes of M2 receptors: Acetylcholinesterase expressions ratio for the adult population. (DOCX)

S12 Table Detailed results of inferential analyzes of M2 receptors expression for the adult population, including the Carotid Sinus Massage Test. (DOCX)

S13 Table Detailed results of inferential analyzes of Acetylcholinesterase expression for the adult population, including the Carotid Sinus Massage Test. (DOCX)

S14 Table Detailed results of inferential analyzes of M2 receptors expression for the adult population, including the HolterTest. (DOCX)

S15 Table Detailed results of inferential analyzes of Acetylcholinesterase expression for the adult population, including the Holter Test. (DOCX)

S16 Table Detailed results of inferential analyzes of M2 receptors expression for the adult population, including the Carotid Sinus Massage Test and Holter Test. (DOCX)

S17 Table Detailed results of inferential analyzes of Acetylcholinesterase expression for the adult population, including the Carotid Sinus Massage Test and Holter Test. (DOCX)

S18 Table Detailed results of inferential analyzes of M2 receptors expression for the pediatric population. (DOCX)

S19 Table Detailed results of inferential analyzes of Acetylcholinesterase expression for the pediatric population. (DOCX)

S20 Table Detailed results of inferential analyzes of M2 receptors: Acetylcholinesterase expressions ratio for the pediactric population. (DOCX)

S1 Fig M_2_R:AchE expressions ratios in all the subjects. (DOCX)

S2 Fig M_2_R:AchE expressions ratios in the adult population. (DOCX)

S3 Fig M_2_R:AchE expressions ratios in the pediatric population. (DOCX)
