# Supplementary material for: Increased expression of blood muscarinic receptors in patients with reflex syncope
Source: PLoS One. 2019 Jul 18;14(7):e0219598. doi: 10.1371/journal.pone.0219598 (PMC6638918; doi:10.1371/journal.pone.0219598)
Supplement: S1 File — (DOCX) [file pone.0219598.s001.docx]

**SUPPORTING INFORMATION**

This appendix has been provided by the authors to give readers additional information about their work

**Supplement to:**

**Increased expression of blood muscarinic receptors in patients with reflex syncope**

**Short title:** Reflex syncope and blood muscarinic receptors

**TABLE OF CONTENTS**

**Study Oversight 4**

Investigators **4**

Authors (exhaustive list and affiliation) **5**

**Additional Methodology Details 7**

Detailled study assessments **7**

*Holter Test* **7**

*Carotid Sinus Massage Test* **7**

**Genes sequence for mRNA expression assessments 8**

M_2_ receptor gene (CHRM2) **8**

AchE gene (ACHE) **8**

Housekeeping gene 18S ribosomal RNA (18S) **8**

**Detailed data of all subjects 9**

*S1 Table. Detailed data of all subjects* **9**

**Supplementary detailed results 13**

Descriptive analyzes **13**

*S2 Table. Detailed results of descriptive analyzes for the total population* **13**

*S3 Table. Detailed results of descriptive analyzes for the adult population* **14**

*S4 Table. Detailed results of descriptive analyzes for the total population* **15**

*S5 Table. Detailed results of descriptive analyzes for the pediatric population* **16**

Inferential analyzes **17**

*S6 Table. Detailed results of inferential analyzes of M_2_ receptors expression for the total population* **17**

*S7 Table. Detailed results of inferential analyzes of Acetylcholinesterase expression for the total population* **18**

*S8 Table. Detailed results of inferential analyzes of M_2_ receptors: Acetylcholinesterase expressions ratio for the total population* **19**

*S9 Table. Detailed results of inferential analyzes of M_2_ receptors expression for the adult population* **20**

*S10 Table. Detailed results of inferential analyzes of AchE expression for the adult population* **20**

*S11 Table. Detailed results of inferential analyzes of M_2_ receptors: Acetylcholinesterase expressions ratio for the adult population* **22**

*S12 Table. Detailed results of inferential analyzes of M_2_ receptors expression for the adult population, including the Carotid Sinus Massage Test* **23**

*S13 Table. Detailed results of inferential analyzes of Acetylcholinesterase expression for the adult population, including the Carotid Sinus Massage Test* **24**

*S14 Table. Detailed results of inferential analyzes of M_2_ receptors expression for the adult population, including the HolterTest* **25**

*S15 Table. Detailed results of inferential analyzes of Acetylcholinesterase expression for the adult population, including the Holter Test* **26**

*S16 Table. Detailed results of inferential analyzes of M_2_ receptors expression for the adult population, including the Carotid Sinus Massage Test and Holter Test* **27**

*S17 Table. Detailed results of inferential analyzes of Acetylcholinesterase expression for the adult population, including the Carotid Sinus Massage Test and Holter Test* **28**

*S18 Table. Detailed results of inferential analyzes of M_2_ receptors expression for the pediatric population* **29**

*S19 Table. Detailed results of inferential analyzes of Acetylcholinesterase expression for the pediatric population* **30**

*S20 Table. Detailed results of inferential analyzes of M_2_ receptors: Acetylcholinesterase expressions ratio for the pediactric population* **31**

**Supplementary figures (with legends) 32**

*S1 Fig. M_2_R:AchE expressions ratios in all the subjects* **32**

*S2 Fig. M_2_R:AchE expressions ratios in the adult population* **33**

*S5 Fig. M_2_R:AchE expressions ratios in the pediatric population*  **34**

**STUDY OVERSIGHT**

**Investigators**

**Dr. Angelo LIVOLSI**, **coordinator investigator**, *Unit of Cardiopediatrics, University Hospitals of Strasbourg, Strasbourg, France.*

**Pr. Pascal BOUSQUET**, associated investigator, *Laboratory of Neurobiology and Cardiovascular Pharmacology, Federation of Translational Medicine, EA 7296, University of Strasbourg, Strasbourg, France.*

**Dr. Pauline HELMS**, associated investigator, *Unit of Cardiopediatrics, University Hospitals of Strasbourg, Strasbourg, France.*

**Dr. Catherine SCHMIDT-MUTTER**, associated investigator, *Center of Clinical Investigation INSERM 1434, University Hospitals of Strasbourg, Strasbourg, France***.**

**Pr. Gerald ROUL**, associated investigator, *Unit of Cardiology, University Hospitals of Strasbourg, Strasbourg, France.*

**Dr. Florian ZORES**, associated investigator, *Unit of Cardiology, University Hospitals of Strasbourg, Strasbourg, France.*

**Authors (exhaustive list and affiliation)**

**Maxime BEUTELSTETTER***, *Center of Clinical Investigation INSERM 1434, University Hospital*

*of Strasbourg, Strasbourg, France.*

**Angelo LIVOLSI*,** M.D*., Unit of Cardiopediatrics, University Hospital of Strasbourg, Strasbourg, France.*

**Hugues GRENEY**, Ph.D., *Laboratory of Neurobiology and Cardiovascular Pharmacology, Federation of Translational Medicine, EA 7296, University of Strasbourg, Strasbourg, France.*

**Pauline HELMS**, M.D., *Unit of Cardiopediatrics, University Hospital of Strasbourg, Strasbourg, France.*

**Catherine SCHMIDT-MUTTER,** M.D., Ph.D., *Center of Clinical Investigation INSERM 1434, University Hospital of Strasbourg, Strasbourg, France***.**

**Charlie DE MELO**, M.D., Unit of Neonatal Intensive Care, *University Hospital of Strasbourg, Strasbourg, France.*

**Gérald ROUL**, M.D., Ph.D., *Unit of Cardiology, University Hospital of Strasbourg, Strasbourg, France.*

**Florian ZORES**, M.D., *Specialized Medical Group – The Premium, Strasbourg, France.*

**Alexandre BOLLE** Pharm.D., *Center of Clinical Investigation INSERM 1434, University Hospital of Strasbourg, Strasbourg, France.*

**Nassim DALI-YOUCEF**, Pharm.D., Ph.D., Laboratory of Biochemistry and Molecular Biology, *University Hospital of Strasbourg, Strasbourg, France. IGBMC, Department of Functional Genomics and Cancer, Illkirch, France.*

**Magali BEAUGEY**, *Laboratory of Neurobiology and Cardiovascular Pharmacology, Federation of Translational Medicine, EA 7296, University of Strasbourg, Strasbourg, France.*

**Alban SIMON,** *Center of Clinical Investigation INSERM 1434, University Hospital of Strasbourg, Strasbourg, France.*

**Nathalie NIEDERHOFFER**, Ph.D., *Laboratory of Neurobiology and Cardiovascular Pharmacology, Federation of Translational Medicine, EA 7296, University of Strasbourg, Strasbourg, France.*

**Jacques REGNARD**, M.D., Ph.D., *Physiology-Functional Explorations, Regional University Hospital of Besançon, Besançon, France.*

**Malika BOUHADDI**, M.D., Ph.D., *Physiology-Functional Explorations, Regional University Hospital of Besançon, Besançon, France.*

**Chris ADAMOPOULOS**, M.D., Ph.D., *Unit of Cardiopediatrics, University Hospital of Strasbourg, Strasbourg, France.*

**Mickael SCHAEFFER**, *Department of Public Health, methods in clinical research, University of Strasbourg, Strasbourg, France.*

**Erik SAULEAU**, M.D., Ph.D., *Department of Public Health, methods in clinical research, University of Strasbourg, Strasbourg, France.*

**Pascal BOUSQUET**, M.D., Ph.D., *Laboratory of Neurobiology and Cardiovascular Pharmacology, Federation of Translational Medicine, EA 7296, University of Strasbourg, Strasbourg, France.*

***: First co-authors, equal contribution.**

**ADDITIONAL METHODOLOGY DETAILS**

**Study assessments**

*Holter:*

The Holter electrocardiographic monitoring with assessment of sinus variability (SV) is currently used as the most relevant assessment in studies on severe vagal syncopes in infant and adult. The SV represents the physiological variations of the RR intervals (between 2 QRS), reflecting the influence of vago-sympathic balance on the sinus node. The data of sinus variability are studied in accordance to the European Society of Cardiology and the North American Society of Stimulation and Electrophysiology (1). The indices reflecting the vagal activity in the time domain are primarily the square root of the difference of consecutive normal RR intervals (rMSSD express in ms) and the percentage of consecutive normal RR intervals differing by more than 50ms (PNN50n express in %). Therefore, we considered positive test (important vagal activity), results higher than 10% compared to reference values, i.e., rMSSD > 56ms in males and > 62ms in females; PNN50 > 28% in males and > 29% in females^1,2,3^.

*^1^: Task Force of the European Society of Cardiology and the North American Society of Pacing and Electrophysiology. Heart rate variability. Standards of measurement, physiological interpretation, and clinical use. Circulation. 1996; 93:43-65.*

*^2^: J. Sztajzel, M. Jung, A. Bayes de Luna : Reproducibility and Gender-Related Differences of Heart Rate Variability during All-Day Activity in Young Men and Women. Ann Noninvasive Electrocardiol. 2008;13:270 – 277.*

*^3^: MW. Chapleau and R. Sabharwal : Methods of assessing vagus nerve activity and reflexes. Heart Fail Rev. 2010; doi 10.1007/s10741-010-9174-6.*

*Carotid Sinus Massage Test:*

The Carotid Sinus Massage Test (CSMT) is currently used for the differential diagnosis between carotid sinus hypersensitivity and convulsive syncopes. In this study the CSMT was performed by manually massaging for 5 to 10 seconds the anterior sternocleidomastoid muscle on a motorized inclination table (Genin-France) with continue blood pressure blood monitoring (in accordance with the FinapressTM instructions) and the heart rate (Task Force Monitor, CNSsytem, Graz, Austria). We considered responses as suggestive of exaggerated parasympathetic activity when reduction of heart rate was greater than 10% and/or arterial pressure decreased by more than 20% compared to the baseline*^4, 5,6,7,8^*.

*^4^: Brignole M, Alboni P, Benditt D, Bergfeldt L, Blanc JJ, Bloch Thomsen PE, et al. : Guidelines on management (diagnosis and treatment) of syncope. Eur Heart J. 2001;22:1256e306.*

*^5^: Almquist A, Gornick CC, Benson Jr DW, Dunnigan A, Benditt DG. Carotid sinus hypersensitivity: evaluation of the vaso-depressor component. Circulation.1985;71:927e37.*

*^6^: Kenny RA, Richardson DA, Steen N, Bexton RS, Shaw FE, Bond J. Carotid sinus syndrome: a modifiable risk factor for nonaccidental falls in older adults (SAFE PACE). J Am Coll Cardiol. 2001;38:1491e6.*

*^7^: Benchimol M. and Oliveira-Souza R. : Diagnostic Relevance of the Carotid Sinus Massage During a Head Up Tilt Table Test (HUTT). Arq Bras Cardiol. 2008;90:264 - 267.*

*^8^: Sture Bevegard B., Shepherd J. T. : Circulatory effects of stimulating the carotid arterial stretch receptors in man at rest and during exercise. J Clin Investigation. 1966;45:133 – 142.*

**Genes sequences for mRNA expression assessments:**

M_2_ receptor gene (CHRM2):

**F**: AAGACCCCGTTTCTCCAAGT - **R**: GAGGCAACAGCACTGACTGA

AchE gene (ACHE):

**F**: TGGAACCCCAACCGTGAG - **R**: GTAGAAGCCACCCCCATAGA.

Housekeeping gene 18S ribosomal RNA (18S):

**F**: CCTGCGGCTTAATTTGACTC – **R**: ATGCCAGAGTCTCGTTCGTT

The RT-qPCR is an accurate and sensitive method to quantify gene expression. To reduce factors which can diminish RT-qPCR accuracy (e.g. quality of RNA, cDNA synthesis by reverse transcriptase, PCR amplification efficiencies), reference genes, known as housekeeping genes (HKGs), are used as internal controls for normalizing the relative expression of target genes. In this study, we are used the 18S ribosomal gene for HKG, the same than that which has be used for the previous studies in rabbits and SIDS ^9, 10,11^.

*^9^: Livolsi A, Niederhoffer N, Dali-Youcef N, Rambaud C, Olexa C, Mokni W, et al. Cardiac muscarinic receptor overexpression in sudden infant death syndrome. PloS One. 2010;5(3):e9464.*

*^10^: Livolsi A, Niederhoffer N, Dali-Youcef N, Mokni W, Olexa-Zorn C, Gies J-P, et al. Constitutive Overexpression of Muscarinic Receptors Leads to Vagal Hyperreactivity. PLoS ONE. 2010;5(12).*

*^11^: Adamopoulos C, Greney H, Beutelstetter M, Bousquet P, Livolsi A. Expression of Circulating Muscarinic Receptors in Infants With Severe Idiopathic Life-Threatening Events. JAMA Pediatr. 2016;170(7):707‑8.*

**DETAILED DATA OF ALL SUBJECTS**

| **Subject number**  *1xxx: children*  *2xxx: adults* | **Groupe**  *patient (p)*  *control (c)* | **Age**  *(years)* | **Sexe**  *female (F)*  *male (M)* | **CSMT** | **Holter** | **M2**  *(a.u.)* | **AchE**  *(a.u.)* | **M2/AchE**  *(a.u.)* |
| --- | --- | --- | --- | --- | --- | --- | --- | --- |
| **1001** | c | 4 | F | * | * | 0,188 | 0,171 | 1,100 |
| **1002** | p | 10 | M | * | * | 8,338 | 7,677 | 1,086 |
| **1003** | p | 3 | M | * | * | 0,211 | 0,420 | 0,502 |
| **1009** | p | 8 | F | * | * | 1,221 | 0,915 | 1,335 |
| **1010** | p | 3 | M | * | * | 0,050 | 0,165 | 0,301 |
| **1011** | p | 4 | F | * | * | 0,267 | 0,292 | 0,912 |
| **1013** | p | 13 | M | * | * | 1,004 | 0,523 | 1,918 |
| **1014** | c | 10 | F | * | * | 1,285 | 0,892 | 1,441 |
| **1016** | p | 17 | F | * | * | 1,885 | 1,553 | 1,214 |
| **1017** | p | 3 | F | * | * | 1,787 | 0,735 | 2,430 |
| **1020** | c | 8 | F | * | * | 0,349 | 0,472 | 0,741 |
| **1021** | p | 12 | F | * | * | 0,534 | 0,680 | 0,784 |
| **1023** | p | 16 | M | * | * | 1,452 | 0,848 | 1,711 |
| **1026** | p | 2 | M | * | * | 1,230 | 1,022 | 1,204 |
| **1027** | p | 1 | M | * | * | 0,261 | 0,119 | 2,189 |
| **1028** | p | 13 | F | * | * | 0,676 | 0,533 | 1,268 |
| **1030** | p | 12 | M | * | * | 5,721 | 3,960 | 1,445 |
| **1031** | p | 5 | F | * | * | 0,031 | 0,122 | 0,253 |
| **1033** | p | 8 | F | * | * | 0,258 | 0,336 | 0,768 |
| **1034** | p | 6 | F | * | * | 0,178 | 0,464 | 0,384 |
| **1035** | p | 9 | M | * | * | 0,931 | 0,975 | 0,955 |
| **1037** | p | 9 | M | * | * | 1,186 | 1,095 | 1,083 |
| **1038** | p | 15 | F | * | * | 0,938 | 0,447 | 2,097 |
| **1039** | p | 2 | M | * | * | 0,230 | 0,340 | 0,677 |
| **1040** | p | 2 | F | * | * | 3,507 | 1,818 | 1,929 |
| **1041** | p | 10 | M | * | * | 0,064 | 0,137 | 0,465 |
| **1044** | p | 5 | F | * | * | 1,271 | 1,062 | 1,197 |
| **1045** | p | 15 | M | * | * | 5,904 | 4,471 | 1,321 |
| **1047** | c | 10 | F | * | * | 0,355 | 0,393 | 0,903 |
| **1048** | p | 11 | F | * | * | 1,108 | 1,034 | 1,072 |
| **1050** | p | 8 | F | * | * | 1,708 | 1,461 | 1,170 |
| **1053** | p | 9 | F | * | * | 0,244 | 0,649 | 0,375 |
| **1054** | p | 15 | M | * | * | 0,169 | 0,554 | 0,305 |
| **1059** | p | 10 | F | * | * | 1,207 | 0,738 | 1,637 |
| **1064** | c | 2 | M | * | * | 1,076 | 0,700 | 1,536 |
| **1066** | c | 4 | M | * | * | 0,235 | 0,423 | 0,555 |
| **1069** | c | 8 | M | * | * | 0,234 | 0,206 | 1,137 |
| **1071** | c | 11 | M | * | * | 0,156 | 0,357 | 0,437 |
| **1072** | c | 13 | F | * | * | 1,063 | 0,791 | 1,343 |
| **1074** | p | 13 | M | * | * | 1,317 | 0,813 | 1,620 |
| **1075** | c | 2 | F | * | * | 1,566 | 1,058 | 1,480 |
| **1077** | c | 17 | M | * | * | 0,172 | 0,154 | 1,119 |
| **1078** | c | 7 | F | * | * | 0,243 | 0,354 | 0,688 |
| **1079** | p | 13 | M | * | * | 0,336 | 0,293 | 1,147 |
| **1080** | c | 7 | M | * | * | 8,858 | 3,819 | 2,320 |
| **1084** | c | 5 | M | * | * | 0,061 | 1,603 | 0,038 |
| **1085** | p | 1 | M | * | * | 2,203 | 2,744 | 0,803 |
| **1086** | p | 13 | F | * | * | 0,863 | 0,210 | 4,099 |
| **1087** | c | 13 | M | * | * | 0,207 | 0,857 | 0,242 |
| **1088** | p | 14 | F | * | * | 0,818 | 0,514 | 1,592 |
| **1089** | p | 14 | F | * | * | 0,286 | 0,555 | 0,515 |
| **1090** | p | 3 | F | * | * | 10,757 | 4,355 | 2,470 |
| **1092** | c | 4 | M | * | * | 0,076 | 2,002 | 0,038 |
| **1093** | c | 6 | M | * | * | 0,541 | 0,357 | 1,515 |
| **1094** | p | 9 | M | * | * | 2,477 | 1,966 | 1,260 |
| **1095** | c | 8 | M | * | * | 0,413 | 0,366 | 1,128 |
| **1097** | c | 6 | M | * | * | 0,142 | 0,359 | 0,396 |
| **1098** | c | 7 | M | * | * | 1,394 | 1,326 | 1,051 |
| **1099** | c | 9 | F | * | * | 1,574 | 1,254 | 1,255 |
| **2001** | c | 34 | M | Negative | Negative | 2,816 | 2,737 | 1,029 |
| **2003** | c | 29 | M | Negative | Negative | 0,480 | 0,494 | 0,971 |
| **2004** | c | 22 | F | Positive | Negative | 0,466 | 0,610 | 0,764 |
| **2005** | c | 41 | F | Negative | Negative | 2,065 | 2,062 | 1,002 |
| **2007** | p | 18 | M | Negative | Positive | 2,357 | 2,179 | 1,082 |
| **2009** | c | 24 | F | Negative | Negative | 0,073 | 0,158 | 0,461 |
| **2010** | c | 27 | M | Positive | Negative | 1,031 | 1,373 | 0,751 |
| **2011** | c | 26 | M | Positive | Negative | 0,472 | 0,643 | 0,734 |
| **2012** | c | 22 | M | Positive | Negative | 0,082 | 0,362 | 0,225 |
| **2013** | c | 26 | M | Positive | Negative | 0,534 | 0,649 | 0,822 |
| **2031** | p | 46 | M | Positive | Negative | 2,632 | 4,393 | 0,599 |
| **2033** | c | 42 | F | Negative | Negative | 0,075 | 1,066 | 0,070 |
| **2035** | c | 25 | F | Negative | Negative | 0,069 | 0,081 | 0,849 |
| **2036** | c | 23 | M | Negative | Negative | 0,094 | 0,177 | 0,533 |
| **2037** | c | 25 | M | Negative | Positive | 0,965 | 1,274 | 0,757 |
| **2038** | c | 21 | M | Negative | Positive | 2,144 | 1,962 | 1,093 |
| **2039** | c | 24 | M | Negative | Negative | 0,082 | 0,158 | 0,520 |
| **2040** | p | 39 | F | Positive | Negative | 0,397 | 0,444 | 0,896 |
| **2042** | c | 19 | M | Negative | Negative | 0,040 | 0,209 | 0,191 |
| **2045** | c | 20 | F | Negative | Negative | 0,126 | 0,126 | 1,006 |
| **2047** | p | 29 | F | Negative | Negative | 8,016 | 5,332 | 1,504 |
| **2050** | c | 25 | M | Negative | Positive | 0,059 | 0,164 | 0,359 |
| **2051** | c | 24 | M | Negative | Negative | 0,602 | 0,684 | 0,881 |
| **2052** | p | 26 | F | Positive | Negative | 0,119 | 0,224 | 0,530 |
| **2053** | p | 21 | F | Positive | Negative | 5,514 | 4,227 | 1,305 |
| **2054** | p | 23 | F | Negative | Positive | 0,089 | 0,252 | 0,351 |
| **2055** | c | 23 | F | Negative | Negative | 2,561 | 1,882 | 1,361 |
| **2056** | p | 24 | F | Positive | Negative | 1,579 | 1,143 | 1,382 |
| **2057** | c | 23 | F | Negative | Negative | 0,085 | 0,145 | 0,587 |
| **2058** | p | 39 | M | Positive | Negative | 2,958 | 2,055 | 1,439 |
| **2059** | p | 23 | F | Negative | Negative | 0,027 | 0,133 | 0,205 |
| **2061** | p | 32 | F | Positive | Negative | 3,107 | 2,329 | 1,334 |
| **2064** | p | 40 | M | Negative | Negative | 0,041 | 0,414 | 0,099 |
| **2065** | c | 23 | M | Negative | Positive | 0,128 | 0,277 | 0,462 |
| **2066** | p | 37 | F | Negative | Negative | 0,112 | 0,202 | 0,557 |
| **2067** | c | 20 | M | Negative | Negative | 0,140 | 0,277 | 0,505 |
| **2068** | c | 18 | M | Negative | Positive | 1,682 | 1,207 | 1,393 |
| **2069** | c | 30 | M | Negative | Negative | 0,138 | 0,164 | 0,845 |
| **2070** | c | 23 | M | Negative | Negative | 0,728 | 0,849 | 0,858 |
| **2071** | c | 22 | F | Negative | Negative | 0,163 | 0,224 | 0,727 |
| **2072** | c | 21 | M | Positive | Positive | 0,107 | 0,183 | 0,585 |
| **2073** | c | 23 | M | Negative | Negative | 1,658 | 1,193 | 1,390 |
| **2074** | p | 20 | F | Positive | Positive | 2,017 | 1,606 | 1,256 |
| **2075** | p | 31 | F | Negative | Negative | 1,944 | 2,239 | 0,868 |
| **2076** | c | 21 | F | Negative | Negative | 0,049 | 0,164 | 0,301 |
| **2077** | c | 22 | F | Positive | Negative | 0,238 | 0,301 | 0,790 |
| **2078** | c | 26 | F | Positive | Negative | 0,043 | 0,393 | 0,108 |
| **2080** | p | 20 | F | Negative | Positive | 0,765 | 0,578 | 1,325 |
| **2083** | p | 21 | F | Negative | Negative | 0,887 | 0,566 | 1,568 |
| **2085** | p | 26 | M | Positive | Positive | 0,111 | 0,240 | 0,461 |
| **2086** | p | 18 | F | Positive | Negative | 0,797 | 0,679 | 1,174 |
| **2087** | p | 28 | F | Positive | Negative | 1,123 | 1,104 | 1,018 |
| **2089** | p | 23 | F | Positive | Positive | 1,026 | 0,804 | 1,276 |
| **2090** | p | 19 | F | Positive | Negative | 0,084 | 0,079 | 1,068 |
| **2091** | p | 34 | F | Positive | Negative | 0,893 | 0,656 | 1,362 |
| **2092** | p | 21 | F | Positive | Positive | 0,915 | 0,704 | 1,301 |
| **2093** | p | 48 | F | Positive | Positive | 3,546 | 2,873 | 1,234 |
| **2094** | p | 26 | F | Positive | Negative | 0,188 | 0,300 | 0,626 |
| **2095** | p | 25 | M | ** | ** | 0,079 | 0,065 | 1,220 |
| **2096** | p | 23 | F | Positive | Positive | 2,170 | 2,630 | 0,825 |
| **2099** | p | 48 | F | Negative | Negative | 0,820 | 0,753 | 1,089 |
| **2101** | p | 22 | F | Positive | Positive | 1,455 | 1,084 | 1,342 |
| **2102** | p | 23 | F | Positive | Positive | 0,870 | 0,788 | 1,104 |
| **2104** | p | 20 | M | Positive | Positive | 1,879 | 1,569 | 1,198 |
| **2105** | p | 25 | F | Positive | Negative | 1,416 | 1,421 | 0,996 |
| **2106** | p | 22 | F | Positive | Negative | 0,857 | 0,916 | 0,935 |
| **2107** | p | 24 | F | Positive | Negative | 1,332 | 1,423 | 0,936 |
| **2109** | p | 24 | F | Positive | Negative | 2,501 | 2,062 | 1,213 |
| **2111** | p | 28 | F | Positive | Positive | 0,115 | 0,154 | 0,744 |
| **2112** | p | 39 | F | Positive | Negative | 3,360 | 2,856 | 1,177 |
| **2113** | p | 19 | M | Positive | Negative | 0,693 | 0,521 | 1,330 |
| **2114** | p | 23 | F | Positive | Positive | 0,579 | 0,496 | 1,167 |
| **2115** | p | 22 | F | Positive | Negative | 0,050 | 0,163 | 0,310 |
| **2116** | p | 25 | F | Positive | Negative | 2,185 | 2,154 | 1,014 |
| **2118** | p | 21 | F | Positive | Negative | 0,041 | 0,534 | 0,076 |
| **2119** | p | 45 | F | Positive | Positive | 0,908 | 1,085 | 0,837 |
| **2121** | p | 19 | F | Positive | Positive | 0,027 | 0,105 | 0,258 |

*S1 Table. Detailed data of all subjects*

*Subject number: 1xxx = pediatric population, 2xxx = adult population*

*Groupe: p=patient group, c=control group*

*Age: age in enrollment*

*Sexe: F=female, M=male*

*M2: value of M_2_ receptors expression (a.u.)*

*AchE: value of AchE expression (a.u.)*

*M2/AchE: value of ratio of M_2_ and AchE expressions (a.u.)*

**: Not applicable*

***: Not done*

**SUPPLEMENTARY DETAILED RESULTS**

**Descriptive analyzes**

| **Variables** | **Group** | **N** | **Min** | **Q1** | **Median** | **Mean** | **Q3** | **Max** | **SD** | **IQR** |
| --- | --- | --- | --- | --- | --- | --- | --- | --- | --- | --- |
| **Age** | **Patient** | 83 | 1.0 | 10.0 | 19.0 | 18.9 | 24.5 | 48.0 | 11.6 | 14.5 |
|  | **Control** | 53 | 2.0 | 8.0 | 21.0 | 18.0 | 24.0 | 42.0 | 9.7 | 16.0 |
| **M_2_** | **Patient** | 83 | 0.0 | 0.3 | 0.9 | 1.5 | 1.9 | 10.8 | 1.9 | 1.6 |
|  | **Control** | 53 | 0.0 | 0.1 | 0.2 | 0.8 | 1.0 | 8.9 | 1.3 | 0.9 |
| **AchE** | **Patient** | 83 | 0.1 | 0.4 | 0.7 | 1.2 | 1.6 | 7.7 | 1.4 | 1.1 |
|  | **Control** | 53 | 0.1 | 0.2 | 0.4 | 0.8 | 1.1 | 2.3 | 0.8 | 0.9 |
| **M_2_/AchE** | **Patient** | 83 | 0.1 | 0.8 | 1.1 | 1.1 | 1.3 | 4.1 | 0.6 | 0.6 |
|  | **Control** | 53 | 0.0 | 0.5 | 0.8 | 0.8 | 1.1 | 2.3 | 0.5 | 0.6 |

*S2 Table. Detailed results of descriptive analyzes for the total population*

*Variables:*

- *Age: Age of subjects in years*
- *M_2_: value of M_2_ receptors expression*
- *AchE: value of AchE expression*
- *M_2_/AchE: value of ratio of M_2_ and AchE expressions*

*N: number of subject in each group*

*Min: minimum value for each variable*

*Q1: first quartile for each variable*

*Median: estimated numeric median value for each variable*

*Mean: estimated numeric mean value for each variable*

*Q3: third quartile for each variable*

*Max: maximum value for each variable*

*SD: standard deviation for each variable*

*IQR: interquartile range for each variable*

| **Variables** | **Group** | **N** | **Min** | **Q1** | **Median** | **Mean** | **Q3** | **Max** | **SD** | **IQR** |
| --- | --- | --- | --- | --- | --- | --- | --- | --- | --- | --- |
| **Age** | **Patient** | 45 | 18.0 | 21.0 | 24.0 | 27.3 | 31.0 | 48. | 8.6 | 10.0 |
|  | **Control** | 32 | 18.0 | 22.0 | 23.0 | 24.8 | 26.0 | 42.0 | 5.4 | 4.0 |
| **M_2_** | **Patient** | 45 | 0.0 | 0.1 | 0.9 | 1.4 | 2.0 | 8.0 | 1.6 | 1.9 |
|  | **Control** | 32 | 0.0 | 0.1 | 0.2 | 0.6 | 0.8 | 2.8 | 0.8 | 0.7 |
| **AchE** | **Patient** | 45 | 0.1 | 0.4 | 0.8 | 1.3 | 2.1 | 5.3 | 1.2 | 1.6 |
|  | **Control** | 32 | 0.1 | 0.2 | 0.4 | 0.7 | 1.1 | 2.7 | 0.7 | 0.9 |
| **M_2_/AchE** | **Patient** | 45 | 0.1 | 0.7 | 1.1 | 1.0 | 1.3 | 1.6 | 0.4 | 0.5 |
|  | **Control** | 32 | 0.1 | 0.5 | 0.8 | 0.7 | 0.9 | 1.4 | 0.3 | 0.4 |

*S3 Table. Detailed results of descriptive analyzes for the adult population*

*Variables:*

- *Age: Age of subjects in years*
- *M_2_: value of M_2_ receptors expression*
- *AchE: value of AchE expression*
- *M_2_/AchE: value of ratio of M_2_ and AchE expressions*

*N: number of subject in each group*

*Min: minimum value for each variable*

*Q1: first quartile for each variable*

*Median: estimated numeric median value for each variable*

*Mean: estimated numeric mean value for each variable*

*Q3: third quartile for each variable*

*Max: maximum value for each variable*

*SD: standard deviation for each variable*

*IQR: interquartile range for each variable*

| **Variables** | **Group** | **N** | **Min** | **Q1** | **Median** | **Mean** | **Q3** | **Max** | **SD** | **IQR** |
| --- | --- | --- | --- | --- | --- | --- | --- | --- | --- | --- |
| **Age** | **Patient**  **CSMT+** | 34 | 18.0 | 21.2 | 24.0 | 26.9 | 28.0 | 48.0 | 8.3 | 6.8 |
|  | **Control**  **CSMT-** | 24 | 18.0 | 21.8 | 23.0 | 25.1 | 25.0 | 42.0 | 6.1 | 3.2 |
| **M_2_** | **Patient**  **CSMT+** | 34 | 0.0 | 0.4 | 1.0 | 1.4 | 2.1 | 5.5 | 1.3 | 1.7 |
|  | **Control**  **CSMT-** | 24 | 0.0 | 0.1 | 0.1 | 0.7 | 1.1 | 2.8 | 0.9 | 1.1 |
| **AchE** | **Patient**  **CSMT+** | 34 | 0.1 | 0.5 | 1.0 | 1.3 | 1.9 | 4.4 | 1.1 | 1.4 |
|  | **Control**  **CSMT-** | 24 | 0.1 | 0.2 | 0.3 | 0.7 | 1.2 | 2.7 | 0.8 | 1.0 |
| **M_2_/AchE** | **Patient**  **CSMT+** | 34 | 0.1 | 0.8 | 1.1 | 1.0 | 1.3 | 1.4 | 0.4 | 0.4 |
|  | **Control**  **CSMT-** | 24 | 0.1 | 0.5 | 0.8 | 0.8 | 1.0 | 1.4 | 0.4 | 0.5 |

*S4 Table. Detailed results of descriptive analyzes for the total population, including the Carotid Sinus Massage Test*

*Variables:*

- *Age: Age of subjects in years*
- *M_2_: value of M_2_ receptors expression*
- *AchE: value of AchE expression*
- *M_2_/AchE: value of ratio of M_2_ and AchE expressions*
- *CSMT - : Negative Carotid Sinus Massage Test*
- *CSMT + Positive Carotid Sinus Massage Test*

*N: number of subject in each group*

*Min: minimum value for each variable*

*Q1: first quartile for each variable*

*Median: estimated numeric median value for each variable*

*Mean: estimated numeric mean value for each variable*

*Q3: third quartile for each variable*

*Max: maximum value for each variable*

*SD: standard deviation for each variable*

*IQR: interquartile range for each variable*

| **Variables** | **Group** | **N** | **Min** | **Q1** | **Median** | **Mean** | **Q3** | **Max** | **SD** | **IQR** |
| --- | --- | --- | --- | --- | --- | --- | --- | --- | --- | --- |
| **Age** | **Patient** | 38 | 1.0 | 4.2 | 9.0 | 8.8 | 13.0 | 17.0 | 4.8 | 8.8 |
|  | **Control** | 21 | 2.0 | 5.0 | 7.0 | 7.7 | 10.0 | 17.0 | 3.8 | 5.0 |
| **M_2_** | **Patient** | 38 | 0.0 | 0.3 | 1.1 | 1.6 | 1.6 | 10.8 | 2.3 | 1.4 |
|  | **Control** | 21 | 0.1 | 0.2 | 0.3 | 1.0 | 1.1 | 8.9 | 1.9 | 0.9 |
| **AchE** | **Patient** | 38 | 0.1 | 0.4 | 0.7 | 1.2 | 1.1 | 7.7 | 1.5 | 0.7 |
|  | **Control** | 21 | 0.2 | 0.4 | 0.5 | 0.9 | 1.1 | 3.8 | 0.8 | 0.7 |
| **M_2_/AchE** | **Patient** | 38 | 0.3 | 0.8 | 1.2 | 1.2 | 1.6 | 4.1 | 0.8 | 0.8 |
|  | **Control** | 21 | 0.0 | 0.6 | 1.1 | 1.0 | 1.3 | 2.3 | 0.6 | 0.8 |

*S5 Table. Detailed results of descriptive analyzes for the pediatric population*

*Variables:*

- *Age: Age of subjects in years*
- *M_2_: value of M_2_ receptors expression*
- *AchE: value of AchE expression*
- *M_2_/AchE: value of ratio of M_2_ and AchE expressions*

*N: number of subject in each group*

*Min: minimum value for each variable*

*Q1: first quartile for each variable*

*Median: estimated numeric median value for each variable*

*Mean: estimated numeric mean value for each variable*

*Q3: third quartile for each variable*

*Max: maximum value for each variable*

*SD: standard deviation for each variable*

*IQR: interquartile range for each variable*

**Inferential analyzes**

|  | **Mean** | **Sd** | **2.5%** | **25%** | **50%** | **75%** | **97.5%** |
| --- | --- | --- | --- | --- | --- | --- | --- |
| **pat conm** | 0.38 | 0.15 | 0.09 | 0.28 | 0.38 | 0.48 | 0.68 |
| **RR pat conm** | 1.48 | 0.23 | 1.09 | 1.32 | 1.46 | 1.62 | 1.98 |
| **Prob pat conm** | 0.99 | 0.07 | 1.00 | 1.00 | 1.00 | 1.00 | 1.00 |

*S6 Table. Detailed results of inferential analyzes of M_2_ receptors expression for the total population*

***pat conm****: variables; pat=patients, conm=controls modality (reference for the estimation)*

***RR pat conm****: relative risk associated with the variable pat (patient) compared to the reference conm (controls modality)*

***Prob pat conm****: probability that the relative risk RR is higher in pat (patients) compared to the reference conm (controls modality)*

*Summary of the posterior distribution (McMC) of each parameter:*

***Mean:*** *mean of the distribution*

***Sd:*** *standard deviation of the distribution*

***2.5%:*** *2.5th percentile of the distribution*

***25%:*** *25th percentile of the distribution*

***50%:*** *50th percentile or median of the distribution*

***75%:*** *75th percentile of the distribution*

***97.5%:*** *97.5th percentile of the distribution*

|  | **mean** | | **sd** | | **2.5%** | | **25%** | | **50%** | | **75%** | | **97.5%** | |  |
| --- | --- | --- | --- | --- | --- | --- | --- | --- | --- | --- | --- | --- | --- | --- | --- |
| **pat conm** | | 0.26 | | 0.14 | | -0.00 | | 0.17 | | 0.26 | | 0.35 | | 0.53 | |
| **RR pat conm** | | 1.31 | | 0.18 | | 1.00 | | 1.18 | | 1.29 | | 1.42 | | 1.70 | |
| **Prob pat conm** | | 0.97 | | 0.16 | | 0.00 | | 1.00 | | 1.00 | | 1.00 | | 1.00 | |

*S7 Table. Detailed results of inferential analyzes of Acetylcholinesterase expression for the total population*

***pat conm****: variables; pat=patients, conm=controls modality (reference for the estimation)*

***RR pat conm****: relative risk associated with the variable pat (patient) compared to the reference conm (controls modality)*

***Prob pat conm****: probability that the relative risk RR is higher in pat (patients) compared to the reference conm (controls modality)*

*Summary of the posterior distribution (McMC) of each parameter:*

***Mean:*** *mean of the distribution*

***Sd:*** *standard deviation of the distribution*

***2.5%:*** *2.5th percentile of the distribution*

***25%:*** *25th percentile of the distribution*

***50%:*** *50th percentile or median of the distribution*

***75%:*** *75th percentile of the distribution*

***97.5%:*** *97.5th percentile of the distribution*

|  | **mean** | **sd** | **2.5%** | **25%** | **50%** | **75%** | **97.5%** |
| --- | --- | --- | --- | --- | --- | --- | --- |
| **pat conm** | 0.30 | 0.10 | 0.10 | 0.23 | 0.30 | 0.37 | 0.50 |
| **RR pat conm** | 1.36 | 0.14 | 1.10 | 1.26 | 1.35 | 1.45 | 1.66 |
| **Prob pat conm** | 1.00 | 0.04 | 1.00 | 1.00 | 1.00 | 1.00 | 1.00 |

*Table S8. Detailed results of inferential analyzes of M_2_ receptors: Acetylcholinesterase expressions ratio for the total population*

***pat conm****: variables; pat=patients, conm=controls modality (reference for the estimation)*

***RR pat conm****: relative risk associated with the variable pat (patient) compared to the reference conm (controls modality)*

***Prob pat conm****: probability that the relative risk RR is higher in pat (patients) compared to the reference conm (controls modality)*

*Summary of the posterior distribution (McMC) of each parameter:*

***Mean:*** *mean of the distribution*

***Sd:*** *standard deviation of the distribution*

***2.5%:*** *2.5th percentile of the distribution*

***25%:*** *25th percentile of the distribution*

***50%:*** *50th percentile or median of the distribution*

***75%:*** *75th percentile of the distribution*

***97.5%:*** *97.5th percentile of the distribution*

|  | **mean** | **sd** | **2.5%** | **25%** | **50%** | **75%** | **97.5%** |
| --- | --- | --- | --- | --- | --- | --- | --- |
| **pat conm** | 0.42 | 0.20 | 0.03 | 0.29 | 0.42 | 0.56 | 0.82 |
| **RR pat conm** | 1.56 | 0.32 | 1.03 | 1.33 | 1.53 | 1.75 | 2.28 |
| **Prob pat conm** | 0.98 | 0.13 | 1.00 | 1.00 | 1.00 | 1.00 | 1.00 |

*S9 Table. Detailed results of inferential analyzes of M_2_ receptors expression for the adult population*

***pat conm****: variables; pat=patients, conm=controls modality (reference for the estimation)*

***RR pat conm****: relative risk associated with the variable pat (patient) compared to the reference conm (controls modality)*

***Prob pat conm****: probability that the relative risk RR is higher in pat (patients) compared to the reference conm (controls modality)*

*Summary of the posterior distribution (McMC) of each parameter:*

***Mean:*** *mean of the distribution*

***Sd:*** *standard deviation of the distribution*

***2.5%:*** *2.5th percentile of the distribution*

***25%:*** *25th percentile of the distribution*

***50%:*** *50th percentile or median of the distribution*

***75%:*** *75th percentile of the distribution*

***97.5%:*** *97.5th percentile of the distribution*

|  | **mean** | **sd** | **2.5%** | **25%** | **50%** | **75%** | **97.5%** |
| --- | --- | --- | --- | --- | --- | --- | --- |
| **pat conm** | 0.34 | 0.18 | -0.01 | 0.22 | 0.34 | 0.46 | 0.70 |
| **RR pat conm** | 1.43 | 0.26 | 0.99 | 1.25 | 1.40 | 1.59 | 2.02 |
| **Prob pat conm** | 0.97 | 0.17 | 0.00 | 1.00 | 1.00 | 1.00 | 1.00 |

*S10 Table. Detailed results of inferential analyzes of AchE expression for the adult population*

***pat conm****: variables; pat=patients, conm=controls modality (reference for the estimation)*

***RR pat conm****: relative risk associated with the variable pat (patient) compared to the reference conm (controls modality)*

***Prob pat conm****: probability that the relative risk RR is higher in pat (patients) compared to the reference conm (controls modality)*

*Summary of the posterior distribution (McMC) of each parameter:*

***Mean:*** *mean of the distribution*

***Sd:*** *standard deviation of the distribution*

***2.5%:*** *2.5th percentile of the distribution*

***25%:*** *25th percentile of the distribution*

***50%:*** *50th percentile or median of the distribution*

***75%:*** *75th percentile of the distribution*

***97.5%:*** *97.5th percentile of the distribution*

|  | **mean** | **sd** | **2.5%** | **25%** | **50%** | **75%** | **97.5%** |
| --- | --- | --- | --- | --- | --- | --- | --- |
| **pat conm** | 0.27 | 0.13 | 0.03 | 0.19 | 0.27 | 0.36 | 0.52 |
| **RR pat conm** | 1.32 | 0.17 | 1.03 | 1.21 | 1.31 | 1.43 | 1.69 |
| **Prob pat conm** | 0.98 | 0.12 | 1.00 | 1.00 | 1.00 | 1.00 | 1.00 |

*S11 Table. Detailed results of inferential analyzes of M_2_ receptors: Acetylcholinesterase expressions ratio for the adult population*

***pat conm****: variables; pat=patients, conm=controls modality (reference for the estimation)*

***RR pat conm****: relative risk associated with the variable pat (patient) compared to the reference conm (controls modality)*

***Prob pat conm****: probability that the relative risk RR is higher in pat (patients) compared to the reference conm (controls modality)*

*Summary of the posterior distribution (McMC) of each parameter:*

***Mean:*** *mean of the distribution*

***Sd:*** *standard deviation of the distribution*

***2.5%:*** *2.5th percentile of the distribution*

***25%:*** *25th percentile of the distribution*

***50%:*** *50th percentile or median of the distribution*

***75%:*** *75th percentile of the distribution*

***97.5%:*** *97.5th percentile of the distribution*

|  | **mean** | **sd** | **2.5%** | **25%** | **50%** | **75%** | **97.5%** |
| --- | --- | --- | --- | --- | --- | --- | --- |
| **pat conm** | 0.52 | 0.23 | 0.07 | 0.36 | 0.52 | 0.67 | 0.97 |
| **RR pat conm** | 1.72 | 0.40 | 1.07 | 1.44 | 1.68 | 1.95 | 2.65 |
| **Prob pat conm** | 0.99 | 0.11 | 1.00 | 1.00 | 1.00 | 1.00 | 1.00 |

*S12 Table. Detailed results of inferential analyzes of M_2_ receptors expression for the adult population, including the Carotid Sinus Massage Test*

***pat conm****: variables; pat=patients, conm=controls modality (reference for the estimation)*

***RR pat conm****: relative risk associated with the variable pat (patient) compared to the reference conm (controls modality)*

***Prob pat conm****: probability that the relative risk RR is higher in pat (patients) compared to the reference conm (controls modality)*

*Summary of the posterior distribution (McMC) of each parameter:*

***Mean:*** *mean of the distribution*

***Sd:*** *standard deviation of the distribution*

***2.5%:*** *2.5th percentile of the distribution*

***25%:*** *25th percentile of the distribution*

***50%:*** *50th percentile or median of the distribution*

***75%:*** *75th percentile of the distribution*

***97.5%:*** *97.5th percentile of the distribution*

|  | **mean** | **sd** | **2.5%** | **25%** | **50%** | **75%** | **97.5%** |
| --- | --- | --- | --- | --- | --- | --- | --- |
| **pat conm** | 0.44 | 0.21 | 0.04 | 0.30 | 0.43 | 0.57 | 0.84 |
| **RR pat conm** | 1.58 | 0.33 | 1.03 | 1.34 | 1.54 | 1.78 | 2.33 |
| **Prob pat conm** | 0.98 | 0.13 | 1.00 | 1.00 | 1.00 | 1.00 | 1.00 |

*S13 Table. Detailed results of inferential analyzes of Acetylcholinesterase expression for the adult population, including the Carotid Sinus Massage Test*

***pat conm****: variables; pat=patients, conm=controls modality (reference for the estimation)*

***RR pat conm****: relative risk associated with the variable pat (patient) compared to the reference conm (controls modality)*

***Prob pat conm****: probability that the relative risk RR is higher in pat (patients) compared to the reference conm (controls modality)*

*Summary of the posterior distribution (McMC) of each parameter:*

***Mean:*** *mean of the distribution*

***Sd:*** *standard deviation of the distribution*

***2.5%:*** *2.5th percentile of the distribution*

***25%:*** *25th percentile of the distribution*

***50%:*** *50th percentile or median of the distribution*

***75%:*** *75th percentile of the distribution*

***97.5%:*** *97.5th percentile of the distribution*

|  | **mean** | **sd** | **2.5%** | **25%** | **50%** | **75%** | **97.5%** |
| --- | --- | --- | --- | --- | --- | --- | --- |
| **pat conm** | 0.52 | 0.26 | 0.00 | 0.35 | 0.53 | 0.70 | 1.03 |
| **RR pat conm** | 1.75 | 0.46 | 1.00 | 1.42 | 1.69 | 2.02 | 2.81 |
| **Prob pat conm** | 0.97 | 0.16 | 1.00 | 1.00 | 1.00 | 1.00 | 1.00 |

*S14 Table. Detailed results of inferential analyzes of M_2_ receptors expression for the adult population, including the HolterTest*

***pat conm****: variables; pat=patients, conm=controls modality (reference for the estimation)*

***RR pat conm****: relative risk associated with the variable pat (patient) compared to the reference conm (controls modality)*

***Prob pat conm****: probability that the relative risk RR is higher in pat (patients) compared to the reference conm (controls modality)*

*Summary of the posterior distribution (McMC) of each parameter:*

***Mean:*** *mean of the distribution*

***Sd:*** *standard deviation of the distribution*

***2.5%:*** *2.5th percentile of the distribution*

***25%:*** *25th percentile of the distribution*

***50%:*** *50th percentile or median of the distribution*

***75%:*** *75th percentile of the distribution*

***97.5%:*** *97.5th percentile of the distribution*

|  | **mean** | **sd** | **2.5%** | **25%** | **50%** | **75%** | **97.5%** |
| --- | --- | --- | --- | --- | --- | --- | --- |
| **pat conm** | 0.37 | 0.23 | -0.10 | 0.22 | 0.37 | 0.52 | 0.81 |
| **RR pat conm** | 1.48 | 0.34 | 0.91 | 1.24 | 1.45 | 1.69 | 2.26 |
| **Prob pat conm** | 0.94 | 0.24 | 0.00 | 1.00 | 1.00 | 1.00 | 1.00 |

*S15 Table. Detailed results of inferential analyzes of Acetylcholinesterase expression for the adult population, including the Holter Test*

***pat conm****: variables; pat=patients, conm=controls modality (reference for the estimation)*

***RR pat conm****: relative risk associated with the variable pat (patient) compared to the reference conm (controls modality)*

***Prob pat conm****: probability that the relative risk RR is higher in pat (patients) compared to the reference conm (controls modality)*

*Summary of the posterior distribution (McMC) of each parameter:*

***Mean:*** *mean of the distribution*

***Sd:*** *standard deviation of the distribution*

***2.5%:*** *2.5th percentile of the distribution*

***25%:*** *25th percentile of the distribution*

***50%:*** *50th percentile or median of the distribution*

***75%:*** *75th percentile of the distribution*

***97.5%:*** *97.5th percentile of the distribution*

|  | **mean** | **sd** | **2.5%** | **25%** | **50%** | **75%** | **97.5%** |
| --- | --- | --- | --- | --- | --- | --- | --- |
| **pat conm** | 0.55 | 0.28 | -0.03 | 0.37 | 0.56 | 0.74 | 1.08 |
| **RR pat conm** | 1.80 | 0.50 | 0.97 | 1.44 | 1.75 | 2.09 | 1.98 |
| **Prob pat conm** | 0.99 | 0.07 | 1.00 | 1.00 | 1.00 | 1.00 | 1.00 |

*S16 Table. Detailed results of inferential analyzes of M_2_ receptors expression for the adult population, including the Carotid Sinus Massage Test and Holter Test*

***pat conm****: variables; pat=patients, conm=controls modality (reference for the estimation)*

***RR pat conm****: relative risk associated with the variable pat (patient) compared to the reference conm (controls modality)*

***Prob pat conm****: probability that the relative risk RR is higher in pat (patients) compared to the reference conm (controls modality)*

*Summary of the posterior distribution (McMC) of each parameter:*

***Mean:*** *mean of the distribution*

***Sd:*** *standard deviation of the distribution*

***2.5%:*** *2.5th percentile of the distribution*

***25%:*** *25th percentile of the distribution*

***50%:*** *50th percentile or median of the distribution*

***75%:*** *75th percentile of the distribution*

***97.5%:*** *97.5th percentile of the distribution*

|  | **mean** | **sd** | **2.5%** | **25%** | **50%** | **75%** | **97.5%** |
| --- | --- | --- | --- | --- | --- | --- | --- |
| **pat conm** | 0.42 | 0.28 | -0.13 | 0.24 | 0.42 | 0.61 | 0.95 |
| **RR pat conm** | 1.58 | 0.44 | 0.88 | 1.27 | 1.53 | 1.83 | 2.58 |
| **Prob pat conm** | 0.94 | 0.25 | 0.00 | 1.00 | 1.00 | 1.00 | 1.00 |

*S17 Table. Detailed results of inferential analyzes of Acetylcholinesterase expression for the adult population, including the Carotid Sinus Massage Test and Holter Test*

***pat conm****: variables; pat=patients, conm=controls modality (reference for the estimation)*

***RR pat conm****: relative risk associated with the variable pat (patient) compared to the reference conm (controls modality)*

***Prob pat conm****: probability that the relative risk RR is higher in pat (patients) compared to the reference conm (controls modality)*

*Summary of the posterior distribution (McMC) of each parameter:*

***Mean:*** *mean of the distribution*

***Sd:*** *standard deviation of the distribution*

***2.5%:*** *2.5th percentile of the distribution*

***25%:*** *25th percentile of the distribution*

***50%:*** *50th percentile or median of the distribution*

***75%:*** *75th percentile of the distribution*

***97.5%:*** *97.5th percentile of the distribution*

|  | **mean** | **sd** | **2.5%** | **25%** | **50%** | **75%** | **97.5%** |
| --- | --- | --- | --- | --- | --- | --- | --- |
| **pat conm** | 0.30 | 0.24 | -0.15 | 0.14 | 0.30 | 0.46 | 0.78 |
| **RR pat conm** | 1.40 | 0.34 | 0.86 | 1.15 | 1.35 | 1.59 | 2.18 |
| **Prob pat conm** | 0.90 | 0.29 | 0.00 | 1.00 | 1.00 | 1.00 | 1.00 |

*S18 Table. Detailed results of inferential analyzes of M_2_ receptors expression for the pediatric population*

***pat conm****: variables; pat=patients, conm=controls modality (reference for the estimation)*

***RR pat conm****: relative risk associated with the variable pat (patient) compared to the reference conm (controls modality)*

***Prob pat conm****: probability that the relative risk RR is higher in pat (patients) compared to the reference conm (controls modality)*

*Summary of the posterior distribution (McMC) of each parameter:*

***Mean:*** *mean of the distribution*

***Sd:*** *standard deviation of the distribution*

***2.5%:*** *2.5th percentile of the distribution*

***25%:*** *25th percentile of the distribution*

***50%:*** *50th percentile or median of the distribution*

***75%:*** *75th percentile of the distribution*

***97.5%:*** *97.5th percentile of the distribution*

|  | **mean** | **sd** | **2.5%** | **25%** | **50%** | **75%** | **97.5%** |
| --- | --- | --- | --- | --- | --- | --- | --- |
| **pat conm** | 0.13 | 0.21 | -0.26 | -0.01 | 0.13 | 0.27 | 0.55 |
| **RR pat conm** | 1.17 | 0.25 | 0.77 | 0.99 | 1.14 | 1.31 | 1.73 |
| **Prob pat conm** | 0.74 | 0.44 | 0.00 | 0.00 | 1.00 | 1.00 | 1.00 |

*S19 Table. Detailed results of inferential analyzes of Acetylcholinesterase expression for the pediatric population*

***pat conm****: variables; pat=patients, conm=controls modality (reference for the estimation)*

***RR pat conm****: relative risk associated with the variable pat (patient) compared to the reference conm (controls modality)*

***Prob pat conm****: probability that the relative risk RR is higher in pat (patients) compared to the reference conm (controls modality)*

*Summary of the posterior distribution (McMC) of each parameter:*

***Mean:*** *mean of the distribution*

***Sd:*** *standard deviation of the distribution*

***2.5%:*** *2.5th percentile of the distribution*

***25%:*** *25th percentile of the distribution*

***50%:*** *50th percentile or median of the distribution*

***75%:*** *75th percentile of the distribution*

***97.5%:*** *97.5th percentile of the distribution*

|  | **mean** | **sd** | **2.5%** | **25%** | **50%** | **75%** | **97.5%** |
| --- | --- | --- | --- | --- | --- | --- | --- |
| **pat conm** | 0.32 | 0.17 | -0.01 | 0.21 | 0.32 | 0.43 | 0.66 |
| **RR pat conm** | 1.40 | 0.24 | 0.99 | 1.23 | 1.38 | 1.54 | 1.94 |
| **Prob pat conm** | 0.97 | 0.16 | 0.00 | 1.00 | 1.00 | 1.00 | 1.00 |

*S20 Table. Detailed results of inferential analyzes of M_2_ receptors: Acetylcholinesterase* *expressions ratio for the pediactric population*

***pat conm****: variables; pat=patients, conm=controls modality (reference for the estimation)*

***RR pat conm****: relative risk associated with the variable pat (patient) compared to the reference conm (controls modality)*

***Prob pat conm****: probability that the relative risk RR is higher in pat (patients) compared to the reference conm (controls modality)*

*Summary of the posterior distribution (McMC) of each parameter:*

***Mean:*** *mean of the distribution*

***Sd:*** *standard deviation of the distribution*

***2.5%:*** *2.5th percentile of the distribution*

***25%:*** *25th percentile of the distribution*

***50%:*** *50th percentile or median of the distribution*

***75%:*** *75th percentile of the distribution*

***97.5%:*** *97.5th percentile of the distribution*

**SUPPLEMENTARY FIGURES (WITH LEGENDS)**

*S1 Fig. M_2_R:AchE expressions ratios in the pooled groups of subjects*

*Medians of the mRNA M_2_R:AchE expression ratio with 25 and 75 percentiles in box plots based on all subject data, and the probability that M_2_R expression is greater in the patient group than the control group [Pr(patients>controls)], estimated from the posterior distribution in regression models*

*S2 Fig. M_2_R:AchE expressions ratios in the adult group*

*Medians of ratio of mRNA M_2_R:AchE expressions with 25 and 75 percentiles in box plots based on all subject data, and the probability that this ratio is greater in the patients group than in the control group [Pr(patients>controls)] estimated from the posterior distribution in regression models*

*S5 Fig.M_2_R:AchE expressions ratios in the pediatric group*

*Medians of ratio of mRNA M_2_R:AchE expressions with 25 and 75 percentiles in box plots based on all subject data, and the probability that this ratio is greater in the patients group than in the control group [Pr(patients>controls)] estimated from the posterior distribution in regression models*
