# Supplementary material for: Increased expression of blood muscarinic receptors in patients with reflex syncope
Source: PLoS One. 2019 Jul 18;14(7):e0219598. doi: 10.1371/journal.pone.0219598 (PMC6638918; doi:10.1371/journal.pone.0219598)
Supplement: S1 Methods — (DOCX) [file pone.0219598.s002.docx]

**ADDITIONAL METHODOLOGY DETAILS**

**Study assessments**

*Holter:*

The Holter electrocardiographic monitoring with assessment of sinus variability (SV) is currently used as the most relevant assessment in studies on severe vagal syncopes in infant and adult. The SV represents the physiological variations of the RR intervals (between 2 QRS), reflecting the influence of vago-sympathic balance on the sinus node. The data of sinus variability are studied in accordance to the European Society of Cardiology and the North American Society of Stimulation and Electrophysiology (1). The indices reflecting the vagal activity in the time domain are primarily the square root of the difference of consecutive normal RR intervals (rMSSD express in ms) and the percentage of consecutive normal RR intervals differing by more than 50ms (PNN50n express in %). Therefore, we considered positive test (important vagal activity), results higher than 10% compared to reference values, i.e., rMSSD > 56ms in males and > 62ms in females; PNN50 > 28% in males and > 29% in females^1,2,3^.

*^1^: Task Force of the European Society of Cardiology and the North American Society of Pacing and Electrophysiology. Heart rate variability. Standards of measurement, physiological interpretation, and clinical use. Circulation. 1996; 93:43-65.*

*^2^: J. Sztajzel, M. Jung, A. Bayes de Luna : Reproducibility and Gender-Related Differences of Heart Rate Variability during All-Day Activity in Young Men and Women. Ann Noninvasive Electrocardiol. 2008;13:270 – 277.*

*^3^: MW. Chapleau and R. Sabharwal : Methods of assessing vagus nerve activity and reflexes. Heart Fail Rev. 2010; doi 10.1007/s10741-010-9174-6.*

*Carotid Sinus Massage Test:*

The Carotid Sinus Massage Test (CSMT) is currently used for the differential diagnosis between carotid sinus hypersensitivity and convulsive syncopes. In this study the CSMT was performed by manually massaging for 5 to 10 seconds the anterior sternocleidomastoid muscle on a motorized inclination table (Genin-France) with continue blood pressure blood monitoring (in accordance with the FinapressTM instructions) and the heart rate (Task Force Monitor, CNSsytem, Graz, Austria). We considered responses as suggestive of exaggerated parasympathetic activity when reduction of heart rate was greater than 10% and/or arterial pressure decreased by more than 20% compared to the baseline*^4, 5,6,7,8^*.

*^4^: Brignole M, Alboni P, Benditt D, Bergfeldt L, Blanc JJ, Bloch Thomsen PE, et al. : Guidelines on management (diagnosis and treatment) of syncope. Eur Heart J. 2001;22:1256e306.*

*^5^: Almquist A, Gornick CC, Benson Jr DW, Dunnigan A, Benditt DG. Carotid sinus hypersensitivity: evaluation of the vaso-depressor component. Circulation.1985;71:927e37.*

*^6^: Kenny RA, Richardson DA, Steen N, Bexton RS, Shaw FE, Bond J. Carotid sinus syndrome: a modifiable risk factor for nonaccidental falls in older adults (SAFE PACE). J Am Coll Cardiol. 2001;38:1491e6.*

*^7^: Benchimol M. and Oliveira-Souza R. : Diagnostic Relevance of the Carotid Sinus Massage During a Head Up Tilt Table Test (HUTT). Arq Bras Cardiol. 2008;90:264 - 267.*

*^8^: Sture Bevegard B., Shepherd J. T. : Circulatory effects of stimulating the carotid arterial stretch receptors in man at rest and during exercise. J Clin Investigation. 1966;45:133 – 142.*

**Genes sequences for mRNA expression assessments:**

M_2_ receptor gene (CHRM2):

**F**: AAGACCCCGTTTCTCCAAGT - **R**: GAGGCAACAGCACTGACTGA

AchE gene (ACHE):

**F**: TGGAACCCCAACCGTGAG - **R**: GTAGAAGCCACCCCCATAGA.

Housekeeping gene 18S ribosomal RNA (18S):

**F**: CCTGCGGCTTAATTTGACTC – **R**: ATGCCAGAGTCTCGTTCGTT

The RT-qPCR is an accurate and sensitive method to quantify gene expression. To reduce factors which can diminish RT-qPCR accuracy (e.g. quality of RNA, cDNA synthesis by reverse transcriptase, PCR amplification efficiencies), reference genes, known as housekeeping genes (HKGs), are used as internal controls for normalizing the relative expression of target genes. In this study, we are used the 18S ribosomal gene for HKG, the same than that which has be used for the previous studies in rabbits and SIDS ^9, 10,11^.

*^9^: Livolsi A, Niederhoffer N, Dali-Youcef N, Rambaud C, Olexa C, Mokni W, et al. Cardiac muscarinic receptor overexpression in sudden infant death syndrome. PloS One. 2010;5(3):e9464.*

*^10^: Livolsi A, Niederhoffer N, Dali-Youcef N, Mokni W, Olexa-Zorn C, Gies J-P, et al. Constitutive Overexpression of Muscarinic Receptors Leads to Vagal Hyperreactivity. PLoS ONE. 2010;5(12).*

*^11^: Adamopoulos C, Greney H, Beutelstetter M, Bousquet P, Livolsi A. Expression of Circulating Muscarinic Receptors in Infants With Severe Idiopathic Life-Threatening Events. JAMA Pediatr. 2016;170(7):707‑8.*
