# Supplementary material for: Increased expression of blood muscarinic receptors in patients with reflex syncope
Source: PLoS One. 2019 Jul 18;14(7):e0219598. doi: 10.1371/journal.pone.0219598 (PMC6638918; doi:10.1371/journal.pone.0219598)
Supplement: S1 Table — (DOCX) [file pone.0219598.s003.docx]

**S1 Table. Detailed data of all subjects**

| **Subject number**  *1xxx: children*  *2xxx: adults* | **Groupe**  *patient (p)*  *control (c)* | **Age**  *(years)* | **Sexe**  *female (F)*  *male (M)* | **CSMT** | **Holter** | **M2**  *(a.u.)* | **AchE**  *(a.u.)* | **M2/AchE**  *(a.u.)* |
| --- | --- | --- | --- | --- | --- | --- | --- | --- |
| **1001** | c | 4 | F | * | * | 0,188 | 0,171 | 1,100 |
| **1002** | p | 10 | M | * | * | 8,338 | 7,677 | 1,086 |
| **1003** | p | 3 | M | * | * | 0,211 | 0,420 | 0,502 |
| **1009** | p | 8 | F | * | * | 1,221 | 0,915 | 1,335 |
| **1010** | p | 3 | M | * | * | 0,050 | 0,165 | 0,301 |
| **1011** | p | 4 | F | * | * | 0,267 | 0,292 | 0,912 |
| **1013** | p | 13 | M | * | * | 1,004 | 0,523 | 1,918 |
| **1014** | c | 10 | F | * | * | 1,285 | 0,892 | 1,441 |
| **1016** | p | 17 | F | * | * | 1,885 | 1,553 | 1,214 |
| **1017** | p | 3 | F | * | * | 1,787 | 0,735 | 2,430 |
| **1020** | c | 8 | F | * | * | 0,349 | 0,472 | 0,741 |
| **1021** | p | 12 | F | * | * | 0,534 | 0,680 | 0,784 |
| **1023** | p | 16 | M | * | * | 1,452 | 0,848 | 1,711 |
| **1026** | p | 2 | M | * | * | 1,230 | 1,022 | 1,204 |
| **1027** | p | 1 | M | * | * | 0,261 | 0,119 | 2,189 |
| **1028** | p | 13 | F | * | * | 0,676 | 0,533 | 1,268 |
| **1030** | p | 12 | M | * | * | 5,721 | 3,960 | 1,445 |
| **1031** | p | 5 | F | * | * | 0,031 | 0,122 | 0,253 |
| **1033** | p | 8 | F | * | * | 0,258 | 0,336 | 0,768 |
| **1034** | p | 6 | F | * | * | 0,178 | 0,464 | 0,384 |
| **1035** | p | 9 | M | * | * | 0,931 | 0,975 | 0,955 |
| **1037** | p | 9 | M | * | * | 1,186 | 1,095 | 1,083 |
| **1038** | p | 15 | F | * | * | 0,938 | 0,447 | 2,097 |
| **1039** | p | 2 | M | * | * | 0,230 | 0,340 | 0,677 |
| **1040** | p | 2 | F | * | * | 3,507 | 1,818 | 1,929 |
| **1041** | p | 10 | M | * | * | 0,064 | 0,137 | 0,465 |
| **1044** | p | 5 | F | * | * | 1,271 | 1,062 | 1,197 |
| **1045** | p | 15 | M | * | * | 5,904 | 4,471 | 1,321 |
| **1047** | c | 10 | F | * | * | 0,355 | 0,393 | 0,903 |
| **1048** | p | 11 | F | * | * | 1,108 | 1,034 | 1,072 |
| **1050** | p | 8 | F | * | * | 1,708 | 1,461 | 1,170 |
| **1053** | p | 9 | F | * | * | 0,244 | 0,649 | 0,375 |
| **1054** | p | 15 | M | * | * | 0,169 | 0,554 | 0,305 |
| **1059** | p | 10 | F | * | * | 1,207 | 0,738 | 1,637 |
| **1064** | c | 2 | M | * | * | 1,076 | 0,700 | 1,536 |
| **1066** | c | 4 | M | * | * | 0,235 | 0,423 | 0,555 |
| **1069** | c | 8 | M | * | * | 0,234 | 0,206 | 1,137 |
| **1071** | c | 11 | M | * | * | 0,156 | 0,357 | 0,437 |
| **1072** | c | 13 | F | * | * | 1,063 | 0,791 | 1,343 |
| **1074** | p | 13 | M | * | * | 1,317 | 0,813 | 1,620 |
| **1075** | c | 2 | F | * | * | 1,566 | 1,058 | 1,480 |
| **1077** | c | 17 | M | * | * | 0,172 | 0,154 | 1,119 |
| **1078** | c | 7 | F | * | * | 0,243 | 0,354 | 0,688 |
| **1079** | p | 13 | M | * | * | 0,336 | 0,293 | 1,147 |
| **1080** | c | 7 | M | * | * | 8,858 | 3,819 | 2,320 |
| **1084** | c | 5 | M | * | * | 0,061 | 1,603 | 0,038 |
| **1085** | p | 1 | M | * | * | 2,203 | 2,744 | 0,803 |
| **1086** | p | 13 | F | * | * | 0,863 | 0,210 | 4,099 |
| **1087** | c | 13 | M | * | * | 0,207 | 0,857 | 0,242 |
| **1088** | p | 14 | F | * | * | 0,818 | 0,514 | 1,592 |
| **1089** | p | 14 | F | * | * | 0,286 | 0,555 | 0,515 |
| **1090** | p | 3 | F | * | * | 10,757 | 4,355 | 2,470 |
| **1092** | c | 4 | M | * | * | 0,076 | 2,002 | 0,038 |
| **1093** | c | 6 | M | * | * | 0,541 | 0,357 | 1,515 |
| **1094** | p | 9 | M | * | * | 2,477 | 1,966 | 1,260 |
| **1095** | c | 8 | M | * | * | 0,413 | 0,366 | 1,128 |
| **1097** | c | 6 | M | * | * | 0,142 | 0,359 | 0,396 |
| **1098** | c | 7 | M | * | * | 1,394 | 1,326 | 1,051 |
| **1099** | c | 9 | F | * | * | 1,574 | 1,254 | 1,255 |
| **2001** | c | 34 | M | Negative | Negative | 2,816 | 2,737 | 1,029 |
| **2003** | c | 29 | M | Negative | Negative | 0,480 | 0,494 | 0,971 |
| **2004** | c | 22 | F | Positive | Negative | 0,466 | 0,610 | 0,764 |
| **2005** | c | 41 | F | Negative | Negative | 2,065 | 2,062 | 1,002 |
| **2007** | p | 18 | M | Negative | Positive | 2,357 | 2,179 | 1,082 |
| **2009** | c | 24 | F | Negative | Negative | 0,073 | 0,158 | 0,461 |
| **2010** | c | 27 | M | Positive | Negative | 1,031 | 1,373 | 0,751 |
| **2011** | c | 26 | M | Positive | Negative | 0,472 | 0,643 | 0,734 |
| **2012** | c | 22 | M | Positive | Negative | 0,082 | 0,362 | 0,225 |
| **2013** | c | 26 | M | Positive | Negative | 0,534 | 0,649 | 0,822 |
| **2031** | p | 46 | M | Positive | Negative | 2,632 | 4,393 | 0,599 |
| **2033** | c | 42 | F | Negative | Negative | 0,075 | 1,066 | 0,070 |
| **2035** | c | 25 | F | Negative | Negative | 0,069 | 0,081 | 0,849 |
| **2036** | c | 23 | M | Negative | Negative | 0,094 | 0,177 | 0,533 |
| **2037** | c | 25 | M | Negative | Positive | 0,965 | 1,274 | 0,757 |
| **2038** | c | 21 | M | Negative | Positive | 2,144 | 1,962 | 1,093 |
| **2039** | c | 24 | M | Negative | Negative | 0,082 | 0,158 | 0,520 |
| **2040** | p | 39 | F | Positive | Negative | 0,397 | 0,444 | 0,896 |
| **2042** | c | 19 | M | Negative | Negative | 0,040 | 0,209 | 0,191 |
| **2045** | c | 20 | F | Negative | Negative | 0,126 | 0,126 | 1,006 |
| **2047** | p | 29 | F | Negative | Negative | 8,016 | 5,332 | 1,504 |
| **2050** | c | 25 | M | Negative | Positive | 0,059 | 0,164 | 0,359 |
| **2051** | c | 24 | M | Negative | Negative | 0,602 | 0,684 | 0,881 |
| **2052** | p | 26 | F | Positive | Negative | 0,119 | 0,224 | 0,530 |
| **2053** | p | 21 | F | Positive | Negative | 5,514 | 4,227 | 1,305 |
| **2054** | p | 23 | F | Negative | Positive | 0,089 | 0,252 | 0,351 |
| **2055** | c | 23 | F | Negative | Negative | 2,561 | 1,882 | 1,361 |
| **2056** | p | 24 | F | Positive | Negative | 1,579 | 1,143 | 1,382 |
| **2057** | c | 23 | F | Negative | Negative | 0,085 | 0,145 | 0,587 |
| **2058** | p | 39 | M | Positive | Negative | 2,958 | 2,055 | 1,439 |
| **2059** | p | 23 | F | Negative | Negative | 0,027 | 0,133 | 0,205 |
| **2061** | p | 32 | F | Positive | Negative | 3,107 | 2,329 | 1,334 |
| **2064** | p | 40 | M | Negative | Negative | 0,041 | 0,414 | 0,099 |
| **2065** | c | 23 | M | Negative | Positive | 0,128 | 0,277 | 0,462 |
| **2066** | p | 37 | F | Negative | Negative | 0,112 | 0,202 | 0,557 |
| **2067** | c | 20 | M | Negative | Negative | 0,140 | 0,277 | 0,505 |
| **2068** | c | 18 | M | Negative | Positive | 1,682 | 1,207 | 1,393 |
| **2069** | c | 30 | M | Negative | Negative | 0,138 | 0,164 | 0,845 |
| **2070** | c | 23 | M | Negative | Negative | 0,728 | 0,849 | 0,858 |
| **2071** | c | 22 | F | Negative | Negative | 0,163 | 0,224 | 0,727 |
| **2072** | c | 21 | M | Positive | Positive | 0,107 | 0,183 | 0,585 |
| **2073** | c | 23 | M | Negative | Negative | 1,658 | 1,193 | 1,390 |
| **2074** | p | 20 | F | Positive | Positive | 2,017 | 1,606 | 1,256 |
| **2075** | p | 31 | F | Negative | Negative | 1,944 | 2,239 | 0,868 |
| **2076** | c | 21 | F | Negative | Negative | 0,049 | 0,164 | 0,301 |
| **2077** | c | 22 | F | Positive | Negative | 0,238 | 0,301 | 0,790 |
| **2078** | c | 26 | F | Positive | Negative | 0,043 | 0,393 | 0,108 |
| **2080** | p | 20 | F | Negative | Positive | 0,765 | 0,578 | 1,325 |
| **2083** | p | 21 | F | Negative | Negative | 0,887 | 0,566 | 1,568 |
| **2085** | p | 26 | M | Positive | Positive | 0,111 | 0,240 | 0,461 |
| **2086** | p | 18 | F | Positive | Negative | 0,797 | 0,679 | 1,174 |
| **2087** | p | 28 | F | Positive | Negative | 1,123 | 1,104 | 1,018 |
| **2089** | p | 23 | F | Positive | Positive | 1,026 | 0,804 | 1,276 |
| **2090** | p | 19 | F | Positive | Negative | 0,084 | 0,079 | 1,068 |
| **2091** | p | 34 | F | Positive | Negative | 0,893 | 0,656 | 1,362 |
| **2092** | p | 21 | F | Positive | Positive | 0,915 | 0,704 | 1,301 |
| **2093** | p | 48 | F | Positive | Positive | 3,546 | 2,873 | 1,234 |
| **2094** | p | 26 | F | Positive | Negative | 0,188 | 0,300 | 0,626 |
| **2095** | p | 25 | M | ** | ** | 0,079 | 0,065 | 1,220 |
| **2096** | p | 23 | F | Positive | Positive | 2,170 | 2,630 | 0,825 |
| **2099** | p | 48 | F | Negative | Negative | 0,820 | 0,753 | 1,089 |
| **2101** | p | 22 | F | Positive | Positive | 1,455 | 1,084 | 1,342 |
| **2102** | p | 23 | F | Positive | Positive | 0,870 | 0,788 | 1,104 |
| **2104** | p | 20 | M | Positive | Positive | 1,879 | 1,569 | 1,198 |
| **2105** | p | 25 | F | Positive | Negative | 1,416 | 1,421 | 0,996 |
| **2106** | p | 22 | F | Positive | Negative | 0,857 | 0,916 | 0,935 |
| **2107** | p | 24 | F | Positive | Negative | 1,332 | 1,423 | 0,936 |
| **2109** | p | 24 | F | Positive | Negative | 2,501 | 2,062 | 1,213 |
| **2111** | p | 28 | F | Positive | Positive | 0,115 | 0,154 | 0,744 |
| **2112** | p | 39 | F | Positive | Negative | 3,360 | 2,856 | 1,177 |
| **2113** | p | 19 | M | Positive | Negative | 0,693 | 0,521 | 1,330 |
| **2114** | p | 23 | F | Positive | Positive | 0,579 | 0,496 | 1,167 |
| **2115** | p | 22 | F | Positive | Negative | 0,050 | 0,163 | 0,310 |
| **2116** | p | 25 | F | Positive | Negative | 2,185 | 2,154 | 1,014 |
| **2118** | p | 21 | F | Positive | Negative | 0,041 | 0,534 | 0,076 |
| **2119** | p | 45 | F | Positive | Positive | 0,908 | 1,085 | 0,837 |
| **2121** | p | 19 | F | Positive | Positive | 0,027 | 0,105 | 0,258 |

*Subject number: 1xxx = pediatric population, 2xxx = adult population*

*Groupe: p=patient group, c=control group*

*Age: age in enrollment*

*Sexe: F=female, M=male*

*M2: value of M_2_ receptors expression (a.u.)*

*AchE: value of AchE expression (a.u.)*

*M2/AchE: value of ratio of M_2_ and AchE expressions (a.u.)*

**: Not applicable*

***: Not done*
