# Supplementary material for: Increased expression of blood muscarinic receptors in patients with reflex syncope
Source: PLoS One. 2019 Jul 18;14(7):e0219598. doi: 10.1371/journal.pone.0219598 (PMC6638918; doi:10.1371/journal.pone.0219598)
Supplement: S4 Table — (DOCX) [file pone.0219598.s006.docx]

**S4 Table. Detailed results of descriptive analyzes for the total population, including the Carotid Sinus Massage Test**

| **Variables** | **Group** | **N** | **Min** | **Q1** | **Median** | **Mean** | **Q3** | **Max** | **SD** | **IQR** |
| --- | --- | --- | --- | --- | --- | --- | --- | --- | --- | --- |
| **Age** | **Patient**  **CSMT+** | 34 | 18.0 | 21.2 | 24.0 | 26.9 | 28.0 | 48.0 | 8.3 | 6.8 |
|  | **Control**  **CSMT-** | 24 | 18.0 | 21.8 | 23.0 | 25.1 | 25.0 | 42.0 | 6.1 | 3.2 |
| **M_2_** | **Patient**  **CSMT+** | 34 | 0.0 | 0.4 | 1.0 | 1.4 | 2.1 | 5.5 | 1.3 | 1.7 |
|  | **Control**  **CSMT-** | 24 | 0.0 | 0.1 | 0.1 | 0.7 | 1.1 | 2.8 | 0.9 | 1.1 |
| **AchE** | **Patient**  **CSMT+** | 34 | 0.1 | 0.5 | 1.0 | 1.3 | 1.9 | 4.4 | 1.1 | 1.4 |
|  | **Control**  **CSMT-** | 24 | 0.1 | 0.2 | 0.3 | 0.7 | 1.2 | 2.7 | 0.8 | 1.0 |
| **M_2_/AchE** | **Patient**  **CSMT+** | 34 | 0.1 | 0.8 | 1.1 | 1.0 | 1.3 | 1.4 | 0.4 | 0.4 |
|  | **Control**  **CSMT-** | 24 | 0.1 | 0.5 | 0.8 | 0.8 | 1.0 | 1.4 | 0.4 | 0.5 |

*Variables:*

- *Age: Age of subjects in years*
- *M_2_: value of M_2_ receptors expression*
- *AchE: value of AchE expression*
- *M_2_/AchE: value of ratio of M_2_ and AchE expressions*
- *CSMT - : Negative Carotid Sinus Massage Test*
- *CSMT + Positive Carotid Sinus Massage Test*

*N: number of subject in each group*

*Min: minimum value for each variable*

*Q1: first quartile for each variable*

*Median: estimated numeric median value for each variable*

*Mean: estimated numeric mean value for each variable*

*Q3: third quartile for each variable*

*Max: maximum value for each variable*

*SD: standard deviation for each variable*

*IQR: interquartile range for each variable*
