# Supplementary material for: Increased expression of blood muscarinic receptors in patients with reflex syncope
Source: PLoS One. 2019 Jul 18;14(7):e0219598. doi: 10.1371/journal.pone.0219598 (PMC6638918; doi:10.1371/journal.pone.0219598)
Supplement: S5 Table — (DOCX) [file pone.0219598.s007.docx]

**S5 Table. Detailed results of descriptive analyzes for the pediatric population**

| **Variables** | **Group** | **N** | **Min** | **Q1** | **Median** | **Mean** | **Q3** | **Max** | **SD** | **IQR** |
| --- | --- | --- | --- | --- | --- | --- | --- | --- | --- | --- |
| **Age** | **Patient** | 38 | 1.0 | 4.2 | 9.0 | 8.8 | 13.0 | 17.0 | 4.8 | 8.8 |
|  | **Control** | 21 | 2.0 | 5.0 | 7.0 | 7.7 | 10.0 | 17.0 | 3.8 | 5.0 |
| **M_2_** | **Patient** | 38 | 0.0 | 0.3 | 1.1 | 1.6 | 1.6 | 10.8 | 2.3 | 1.4 |
|  | **Control** | 21 | 0.1 | 0.2 | 0.3 | 1.0 | 1.1 | 8.9 | 1.9 | 0.9 |
| **AchE** | **Patient** | 38 | 0.1 | 0.4 | 0.7 | 1.2 | 1.1 | 7.7 | 1.5 | 0.7 |
|  | **Control** | 21 | 0.2 | 0.4 | 0.5 | 0.9 | 1.1 | 3.8 | 0.8 | 0.7 |
| **M_2_/AchE** | **Patient** | 38 | 0.3 | 0.8 | 1.2 | 1.2 | 1.6 | 4.1 | 0.8 | 0.8 |
|  | **Control** | 21 | 0.0 | 0.6 | 1.1 | 1.0 | 1.3 | 2.3 | 0.6 | 0.8 |

*Variables:*

- *Age: Age of subjects in years*
- *M_2_: value of M_2_ receptors expression*
- *AchE: value of AchE expression*
- *M_2_/AchE: value of ratio of M_2_ and AchE expressions*

*N: number of subject in each group*

*Min: minimum value for each variable*

*Q1: first quartile for each variable*

*Median: estimated numeric median value for each variable*

*Mean: estimated numeric mean value for each variable*

*Q3: third quartile for each variable*

*Max: maximum value for each variable*

*SD: standard deviation for each variable*

*IQR: interquartile range for each variable*
