# Supplementary material for: Increased expression of blood muscarinic receptors in patients with reflex syncope
Source: PLoS One. 2019 Jul 18;14(7):e0219598. doi: 10.1371/journal.pone.0219598 (PMC6638918; doi:10.1371/journal.pone.0219598)
Supplement: S7 Table — (DOCX) [file pone.0219598.s009.docx]

**S7 Table. Detailed results of inferential analyzes of Acetylcholinesterase expression for the total population**

|  | **mean** | | **sd** | | **2.5%** | | **25%** | | **50%** | | **75%** | | **97.5%** | |  |
| --- | --- | --- | --- | --- | --- | --- | --- | --- | --- | --- | --- | --- | --- | --- | --- |
| **pat conm** | | 0.26 | | 0.14 | | -0.00 | | 0.17 | | 0.26 | | 0.35 | | 0.53 | |
| **RR pat conm** | | 1.31 | | 0.18 | | 1.00 | | 1.18 | | 1.29 | | 1.42 | | 1.70 | |
| **Prob pat conm** | | 0.97 | | 0.16 | | 0.00 | | 1.00 | | 1.00 | | 1.00 | | 1.00 | |

***pat conm****: variables; pat=patients, conm=controls modality (reference for the estimation)*

***RR pat conm****: relative risk associated with the variable pat (patient) compared to the reference conm (controls modality)*

***Prob pat conm****: probability that the relative risk RR is higher in pat (patients) compared to the reference conm (controls modality)*

*Summary of the posterior distribution (McMC) of each parameter:*

***Mean:*** *mean of the distribution*

***Sd:*** *standard deviation of the distribution*

***2.5%:*** *2.5th percentile of the distribution*

***25%:*** *25th percentile of the distribution*

***50%:*** *50th percentile or median of the distribution*

***75%:*** *75th percentile of the distribution*

***97.5%:*** *97.5th percentile of the distribution*
