# Supplementary material for: Increased expression of blood muscarinic receptors in patients with reflex syncope
Source: PLoS One. 2019 Jul 18;14(7):e0219598. doi: 10.1371/journal.pone.0219598 (PMC6638918; doi:10.1371/journal.pone.0219598)
Supplement: S16 Table — (DOCX) [file pone.0219598.s018.docx]

**S16 Table. Detailed results of inferential analyzes of M_2_ receptors expression for the adult population, including the Carotid Sinus Massage Test and Holter Test**

|  | **mean** | **sd** | **2.5%** | **25%** | **50%** | **75%** | **97.5%** |
| --- | --- | --- | --- | --- | --- | --- | --- |
| **pat conm** | 0.55 | 0.28 | -0.03 | 0.37 | 0.56 | 0.74 | 1.08 |
| **RR pat conm** | 1.80 | 0.50 | 0.97 | 1.44 | 1.75 | 2.09 | 1.98 |
| **Prob pat conm** | 0.99 | 0.07 | 1.00 | 1.00 | 1.00 | 1.00 | 1.00 |

***pat conm****: variables; pat=patients, conm=controls modality (reference for the estimation)*

***RR pat conm****: relative risk associated with the variable pat (patient) compared to the reference conm (controls modality)*

***Prob pat conm****: probability that the relative risk RR is higher in pat (patients) compared to the reference conm (controls modality)*

*Summary of the posterior distribution (McMC) of each parameter:*

***Mean:*** *mean of the distribution*

***Sd:*** *standard deviation of the distribution*

***2.5%:*** *2.5th percentile of the distribution*

***25%:*** *25th percentile of the distribution*

***50%:*** *50th percentile or median of the distribution*

***75%:*** *75th percentile of the distribution*

***97.5%:*** *97.5th percentile of the distribution*
