# Supplementary material for: Increased expression of blood muscarinic receptors in patients with reflex syncope
Source: PLoS One. 2019 Jul 18;14(7):e0219598. doi: 10.1371/journal.pone.0219598 (PMC6638918; doi:10.1371/journal.pone.0219598)
Supplement: S1 Fig — (DOCX) [file pone.0219598.s023.docx]

**S1 Fig. M_2_R:AchE expressions ratios in the pooled groups of subjects**

*Medians of the mRNA M_2_R:AchE expression ratio with 25 and 75 percentiles in box plots based on all subject data, and the probability that M_2_R expression is greater in the patient group than the control group [Pr(patients>controls)], estimated from the posterior distribution in regression models*
