# Supplementary material for: Increased expression of blood muscarinic receptors in patients with reflex syncope
Source: PLoS One. 2019 Jul 18;14(7):e0219598. doi: 10.1371/journal.pone.0219598 (PMC6638918; doi:10.1371/journal.pone.0219598)
Supplement: S3 Fig — (DOCX) [file pone.0219598.s025.docx]

**S5 Fig.M_2_R:AchE expressions ratios in the pediatric group**

*Medians of ratio of mRNA M_2_R:AchE expressions with 25 and 75 percentiles in box plots based on all subject data, and the probability that this ratio is greater in the patients group than in the control group [Pr(patients>controls)] estimated from the posterior distribution in regression models*
